# Supplementary material for: Direct Amination of Nitroquinoline Derivatives via Nucleophilic Displacement of Aromatic Hydrogen
Source: Molecules. 2021 Mar 25;26(7):1857. doi: 10.3390/molecules26071857 (PMC8036465; doi:10.3390/molecules26071857)
Supplement: Supplementary file 1 [file molecules-26-01857-s001.pdf]

Supporting Information for

# Direct amination of nitroquinoline derivatives via nucleophilic displacement of an aromatic hydrogen

**Jakub Wantulok [1], Daniel Swoboda [1], Jacek E. Nycz [1,*], Maria Książek [2], Joachim Kusz [2], Jan Grzegorz Malecki [1] and Vladimír Kubíček [3]**

1 Institute of Chemistry, Faculty of Science and Technology, University of Silesia in Katowice, ul. Szkolna 9; PL-40007 Katowice, Poland; jakub.wantulok1@gmail.com (J.W.); daniel.swoboda@us.edu.pl (D.S.); jan.malecki@us.edu.pl (J.G.M.)

2 Institute of Physics, Faculty of Science and Technology, Univeristy of Silesia in Katowice, 75 Pułku Piechoty 1a, 41-500 Chorzów, Poland; maria.ksiazek@us.edu.pl (M.K.); joachim.kusz@us.edu.pl (J.K.)

3 Charles University Prague, Faculty of Pharmacy in Hradec Králové, Akademika Heyrovského 1203, 500 05 Hradec Králové, Czech Republic; kubicek@faf.cuni.cz (V.K)

* Correspondence: jacek.nycz@us.edu.pl; Tel.: +48-32-359-1446 (J.N.)

***Table of Contents***

Table 1, 2 and 3 S2-S3

$^{1}$H, $^{13}$C and HMQC; and MS spectra of the compounds S4-S31

**Table S1.** Selected bond lengths (**Å**) and angles (°) for **4b** and **4c**.

| Bond lengths [Å] | | | | | |
|---|---|---|---|---|---|
| **4b** | | **4c** | | | |
| N2—C8 | 1.473(4) | N12—C15 | 1.464(3) | N22—C25 | 1.467(3) |
| C3—Cl1 | 1.733(3) | C18—C110 | 1.533(4) | C28—C210 | 1.542(2) |
| N2—O1 | 1.213(4) | N12—O11 | 1.218(3) | N22—O22 | 1.233(3) |
| N2—O2 | 1.208(3) | N12—O12 | 1.228(3) | N22—O21 | 1.213(3) |
| N1—C9 | 1.365(4) | N11—C12 | 1.316(3) | N21—C22 | 1.316(3) |
| N1—C1 | 1.316(4) | N11—C18A | 1.368(2) | N21—C28A | 1.371(3) |
| Angles (°) | | | | | |
| **4b** | | **4c** | | | |
| N2—C8—C9 | 119.0(2) | N12—C15—C16 | 117.4(2) | N22—C25—C24A | 121.3(2) |
| N2—C8—C7 | 117.7(3) | C14A—C15—N12 | 121.8(2) | C26—C25—N22 | 116.6(2) |
| C2—C3—Cl1 | 120.1(3) | C210—C28—C28A | 122.4(2) | C18A—C18—C110 | 122.4(2) |
| C4—C3—Cl1 | 119.3(2) | C27—C28—C210 | 120.2(2) | C17—C18—C110 | 120.9(2) |
| O2—N2—O1 | 123.7(3) | O12—N12—O11 | 123.5(2) | O21—N22—O22 | 123.5(2) |

**Table S2.** Selected bond lengths (**Å**) and angles (°) for **5a**.

| Bond lengths [Å] | | | | | |
|---|---|---|---|---|---|
| **5a** | | | | | |
| N120—C117 | 1.466(4) | N220—C217 | 1.472(5) | N320—C317 | 1.464(6) |
| N316—C318 | 1.362(5) | N316—C315 | 1.318(5) | N39—C310 | 1.419(4) |
| N216—C215 | 1.317(5) | N216—C219 | 1.359(4) | N29—C210 | 1.414(4) |
| N116—C115 | 1.323(6) | N116—C118 | 1.362(4) | N19—C110 | 1.423(5) |
| Angles (°) | | | | | |
| **5a** | | | | | |
| C118—C117—N120 | 118.5(3) | C110—C117—N120 | 119.3(3) | C110—N19—C19A | 126.8(3) |
| C210—C217—N220 | 120.4(3) | C219—C217—N220 | 116.6(3) | C110—N19—C18A | 124.6(3) |
| C310—C317—N320 | 119.7(3) | C210—N29—C28A | 124.1(3) | C210—N29—C29B | 124.2(3) |
| C318—C317—N320 | 117.3(3) | C310—N39—C39B | 126.4(3) | C310—N39—C38A | 123.4(3) |

**Table S3.** Selected bond lengths (**Å**) and angles (°) for **5b**.

| | | | | | |
|---|---|---|---|---|---|
| Bond lengths [Å] | | | | | |
| **5b** | | | | | |
| C110—N19 | 1.416(4) | C210—N29 | 1.413(4) | | |
| N120—O11 | 1.355(4) | N220—O21 | 1.365(4) | | |
| C117—N120 | 1.304(4) | C217—N220 | 1.297(4) | | |
| C112—O12 | 1.228(4) | C212—O22 | 1.219(4) | | |
| Angles (°) | | | | | |
| **5b** | | | | | |
| N120—C117—C110 | 115.2(3) | C218—C217—N220 | 127.2(3) | C110—N19—C19A | 124.7(2) |
| C118—C117—N120 | 127.3(3) | C210—C217—N220 | 115.4(3) | C110—N19—C18A | 126.3(2) |
| C119—C112—O12 | 121.1(3) | O22—C212—C211 | 122.2(3) | C210—N29—C28A | 126.2(2) |
| C111—C112—O12 | 121.2(3) | O22—C212—C219 | 120.9(3) | C210—N29—C29A | 125.6(2) |

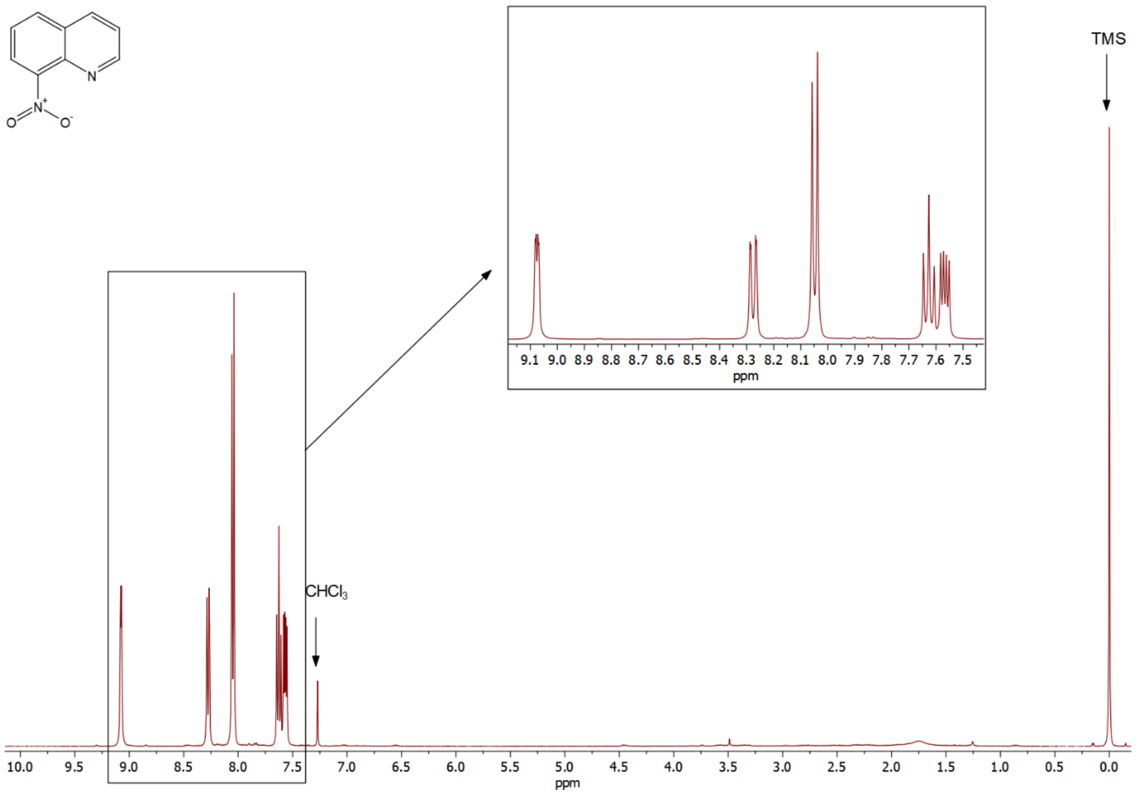


**Figure S1a.** $^{1}$H-NMR ($CDCl_3$; 400.2 MHz) spectrum of the **4a**.

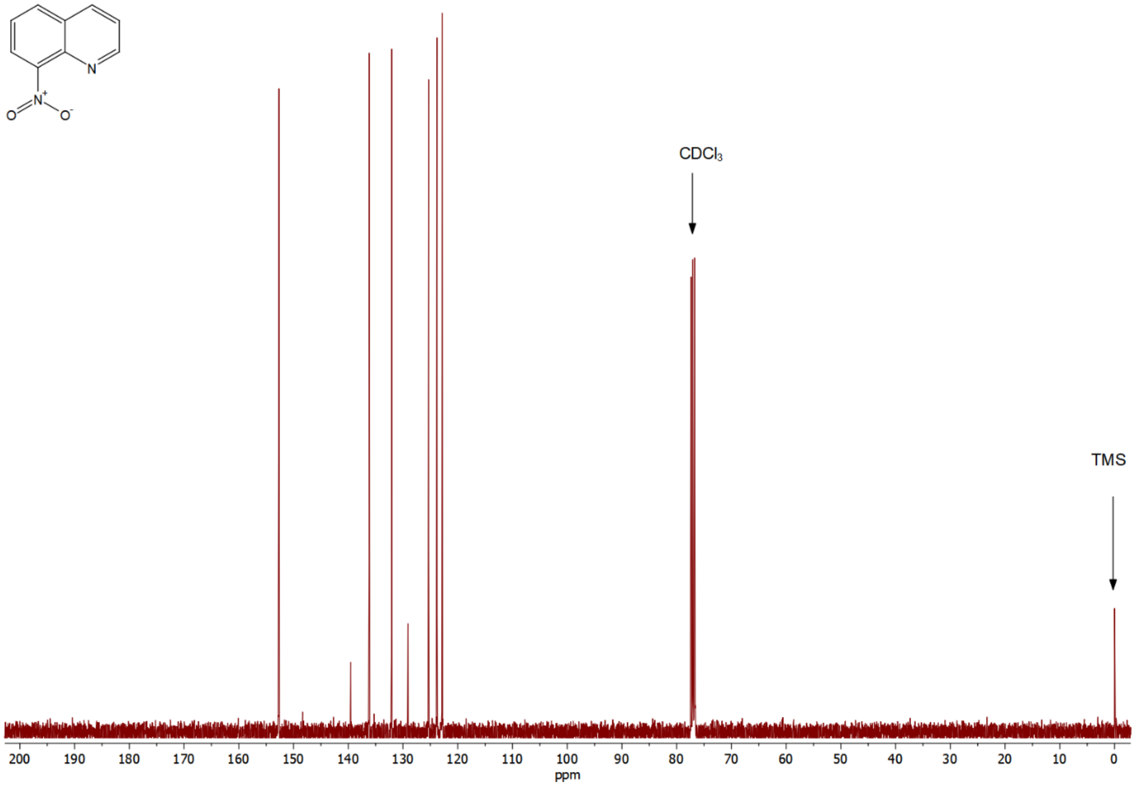


**Fig. S1b.** $^{13}C\{^{1}H\}$ NMR ($CDCl_3$; 100.6 MHz) spectrum of the **4a**.

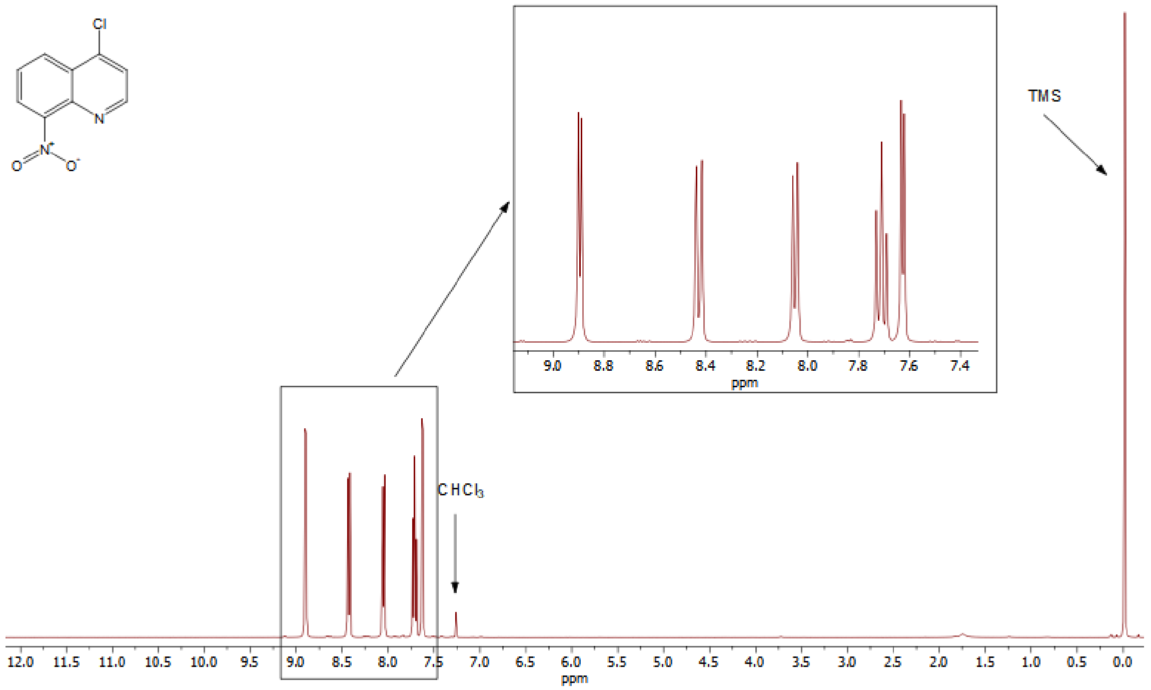


**Fig. S2a.** $^{1}$H NMR ($CDCl_3$; 400.2 MHz) spectrum of the **4b**.

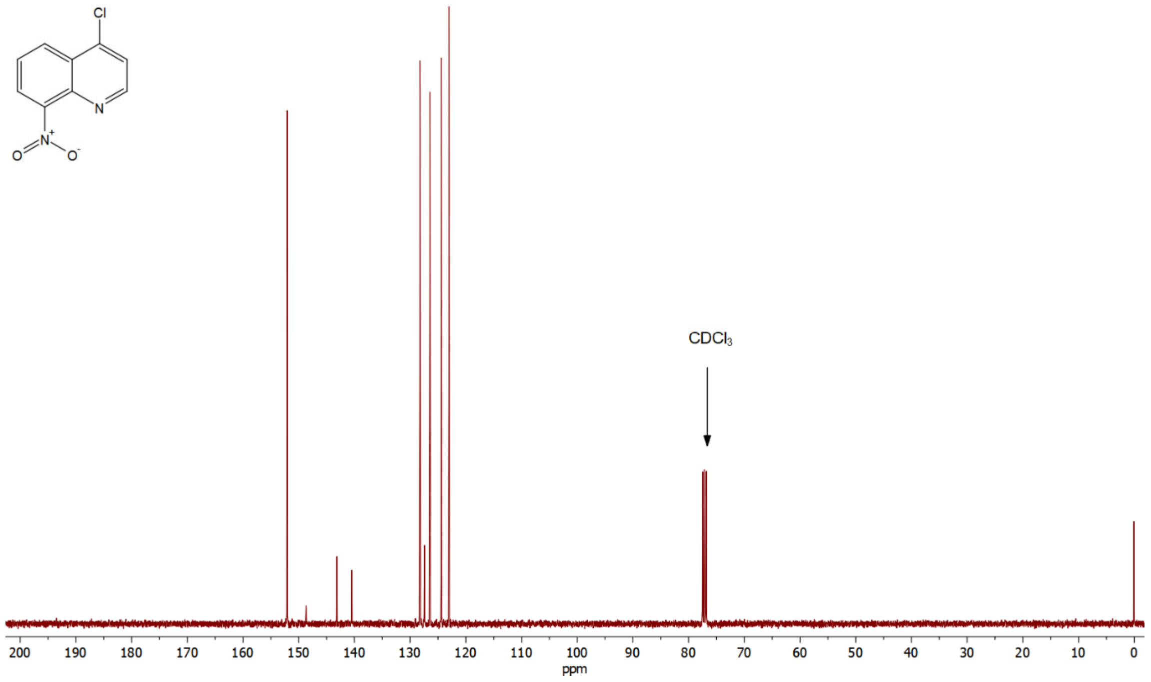


**Fig. S2b.** $^{13}C\{^{1}H\}$ NMR ($CDCl_3$; 100.6 MHz) spectrum of the **4b**.

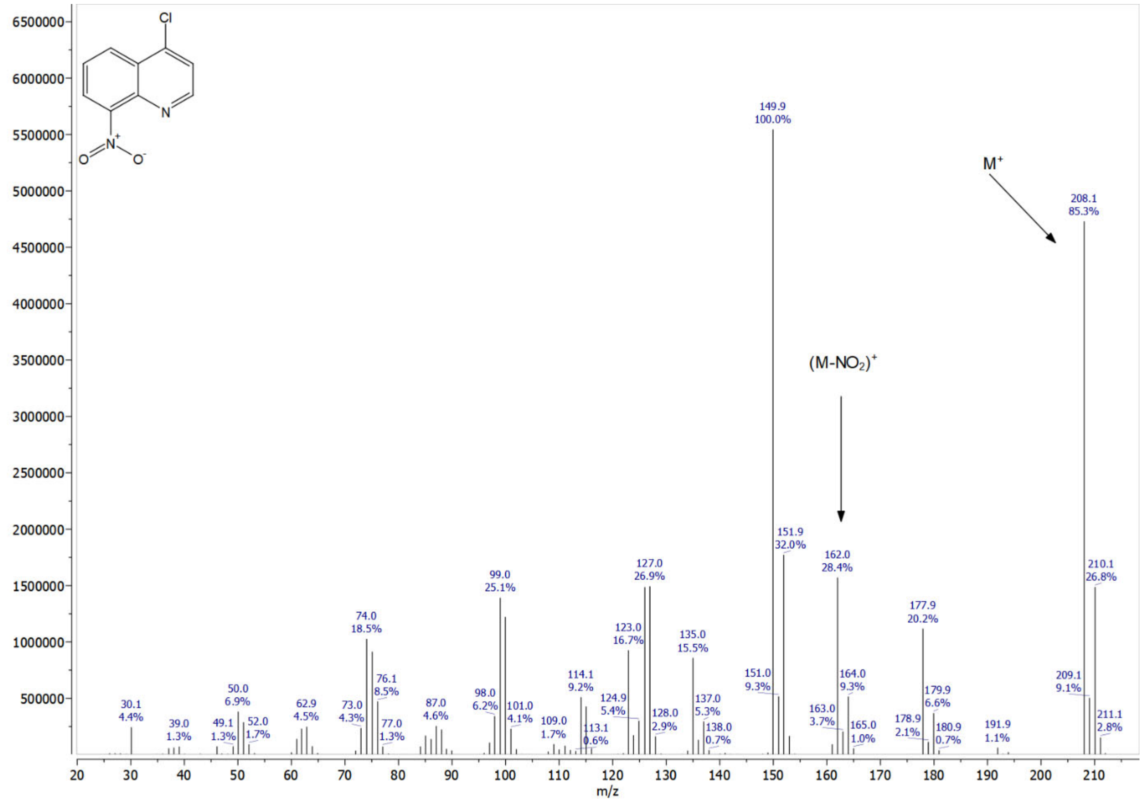


**Fig. S2c.** MS spectrum of the **4b**.

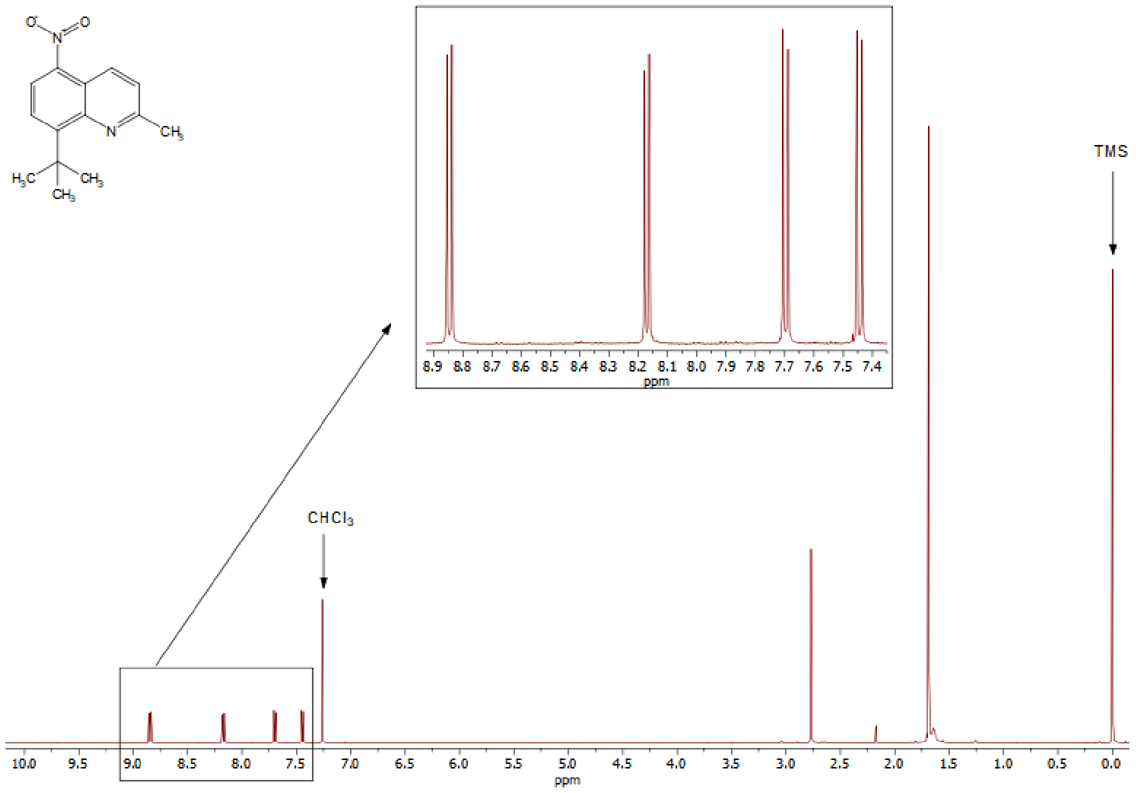


**Fig. S3a.** $^{1}H$ NMR ($CDCl_3$; 500.2 MHz) spectrum of the **4c**.

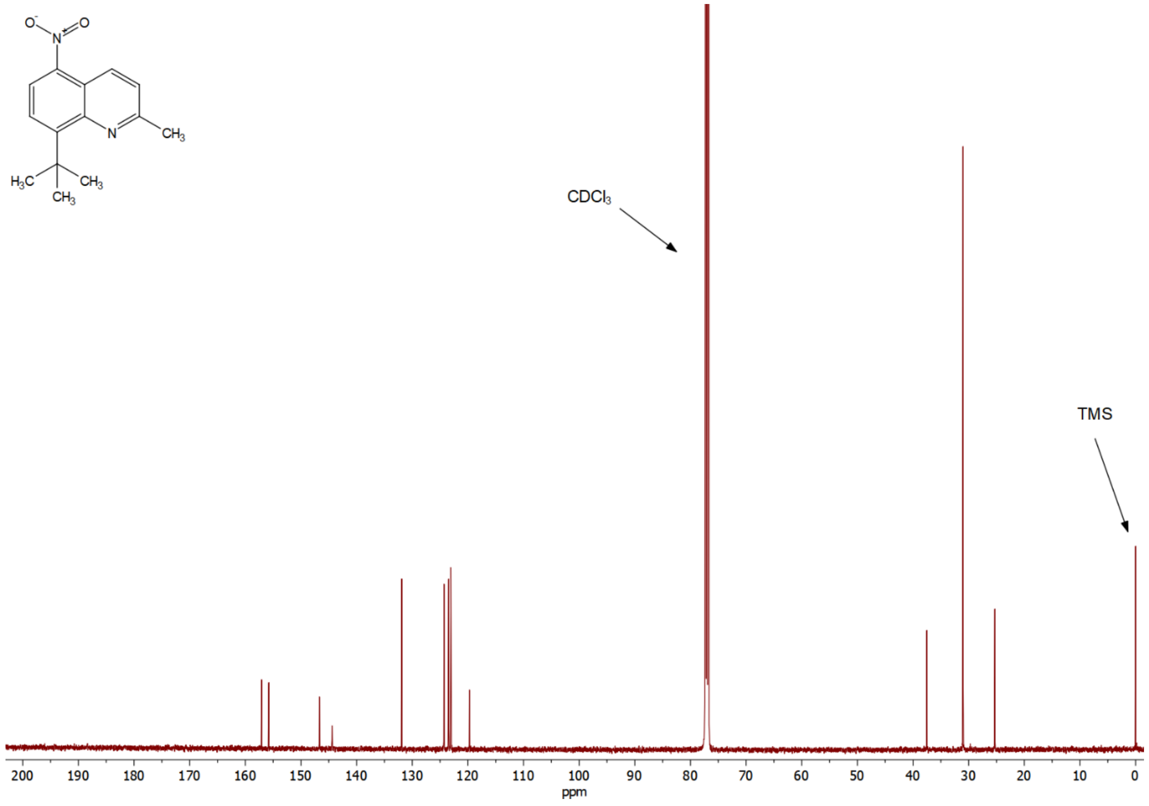


**Fig. S3b.** $^{13}C\{^{1}H\}$ NMR ($CDCl_3$; 125.8 MHz) spectrum of the **4c**.

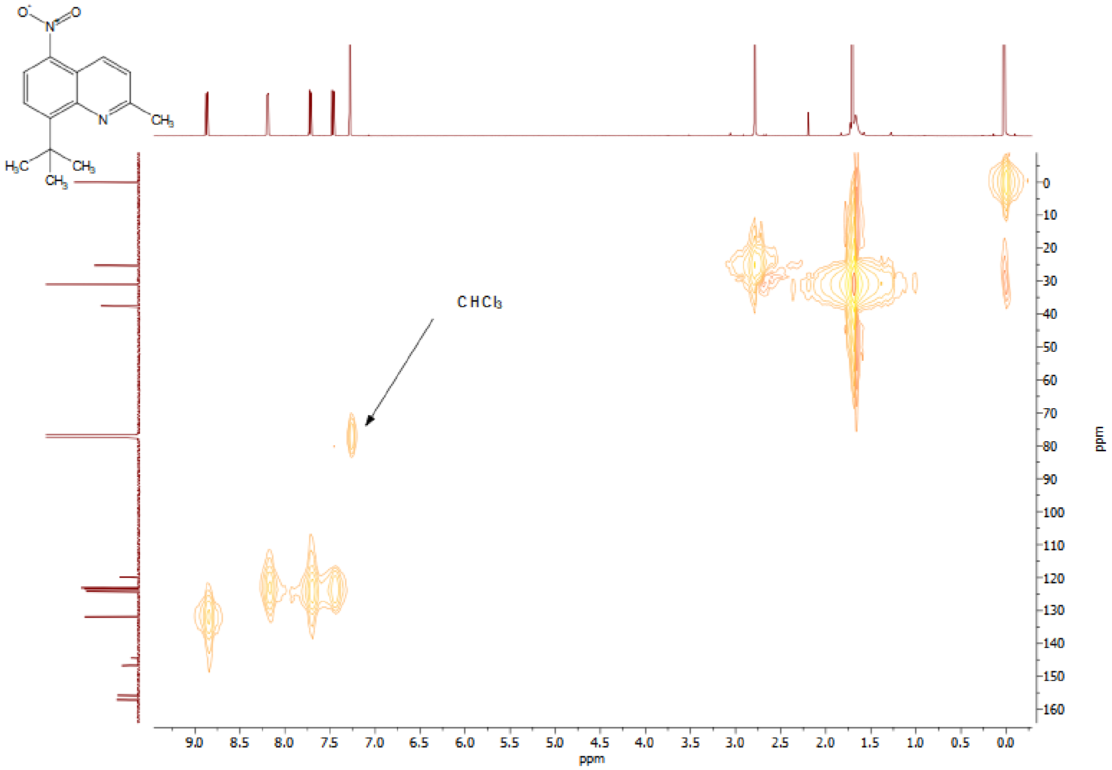


**Fig. S3c.** $^{1}H$ - $^{13}C$ HMQC spectrum of the **4c**.

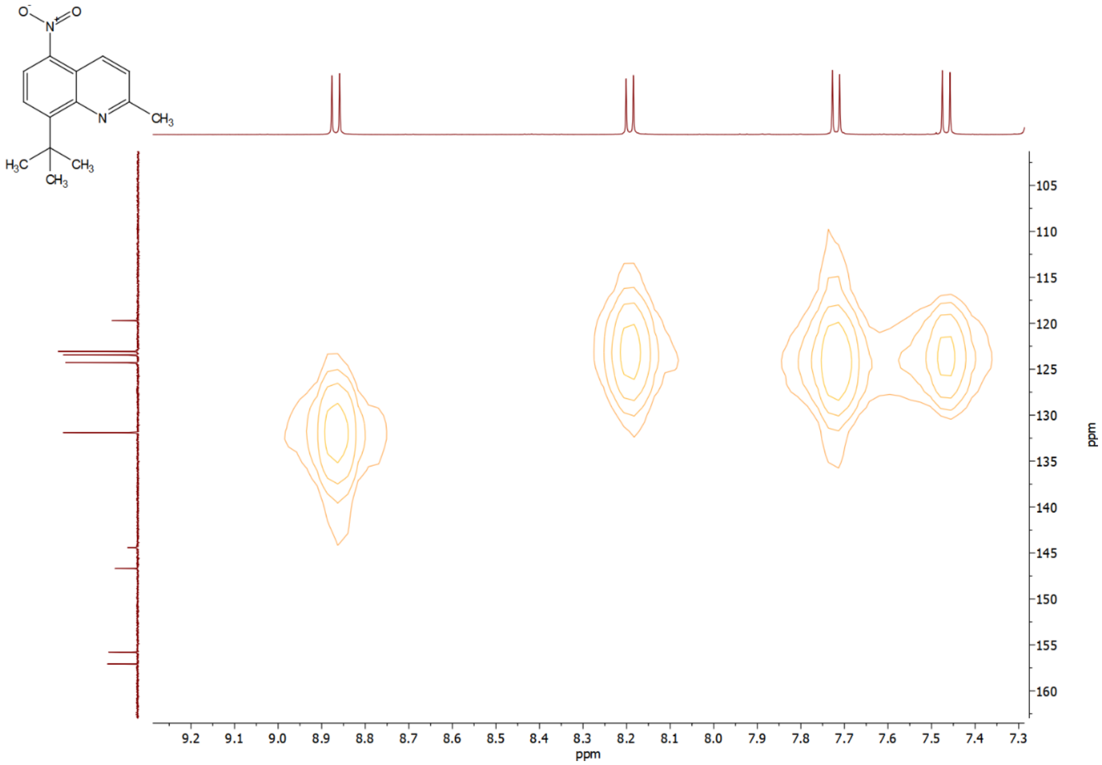


**Fig. S3d.** $^{1}H$ – $^{13}C$ HMQC spectrum (aromatic range) of the **4c**.

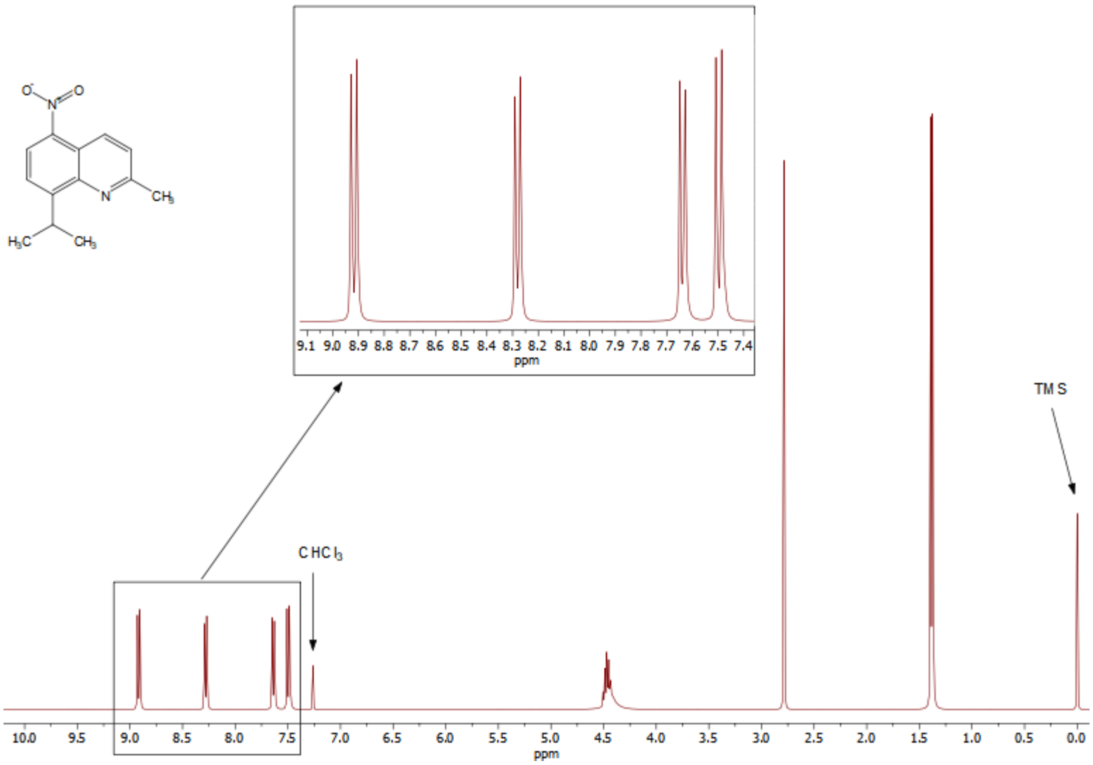


**Fig. S4a.** $^{1}$H NMR ($CDCl_3$; 400.2 MHz) spectrum of the **4d**.

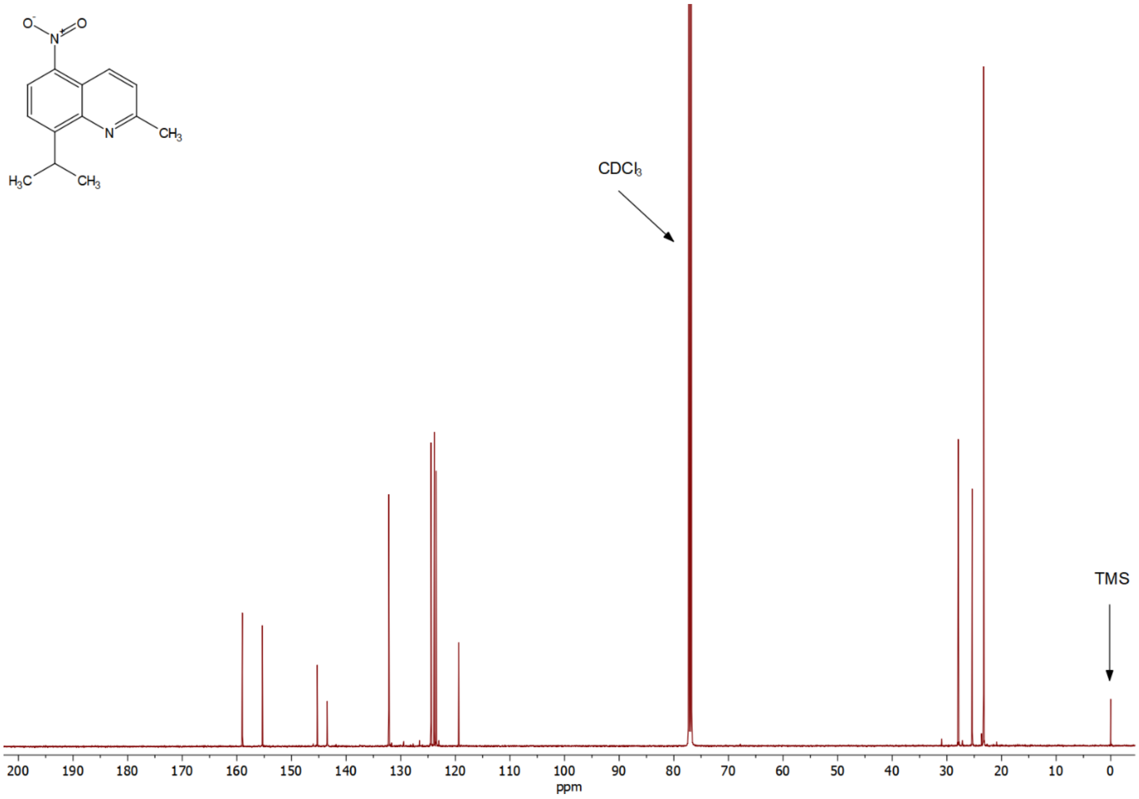


**Fig. S4b.** $^{13}C\{^{1}H\}$ NMR ($CDCl_3$; 125.8 MHz) spectrum of the **4d**.

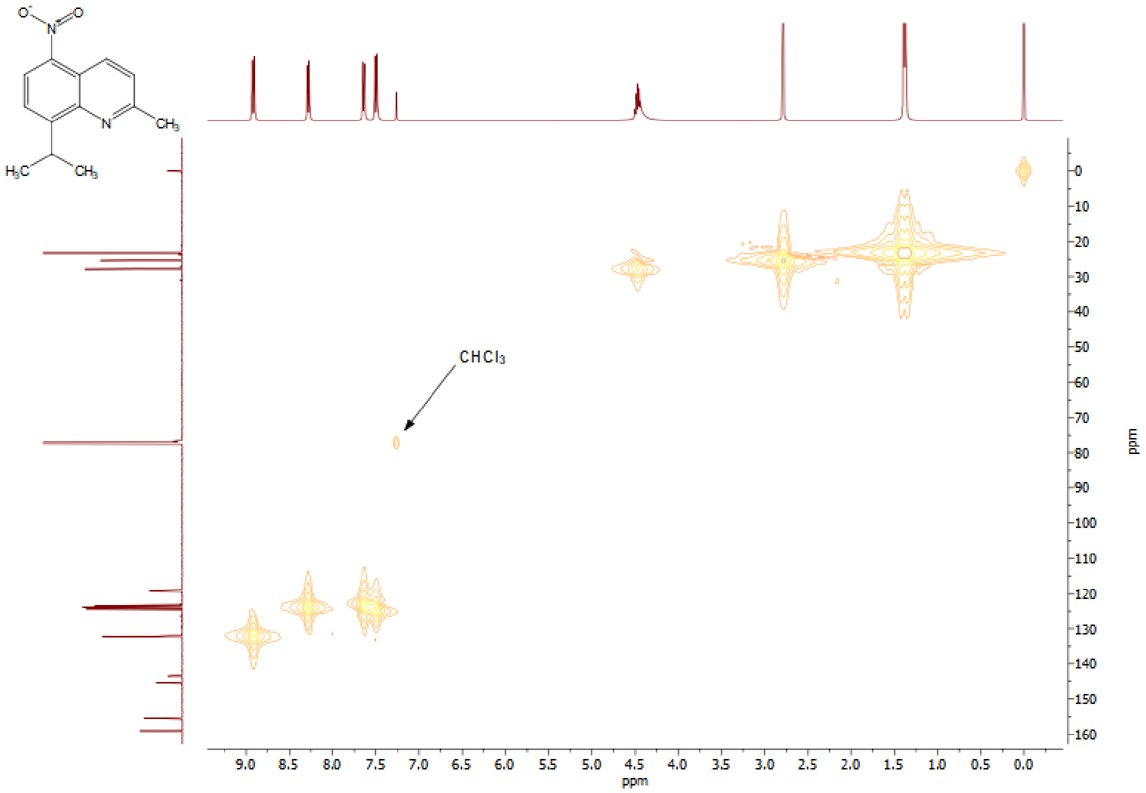


**Fig. S4c.** $^{1}H$ - $^{13}C$ HMQC spectrum of the **4d**.

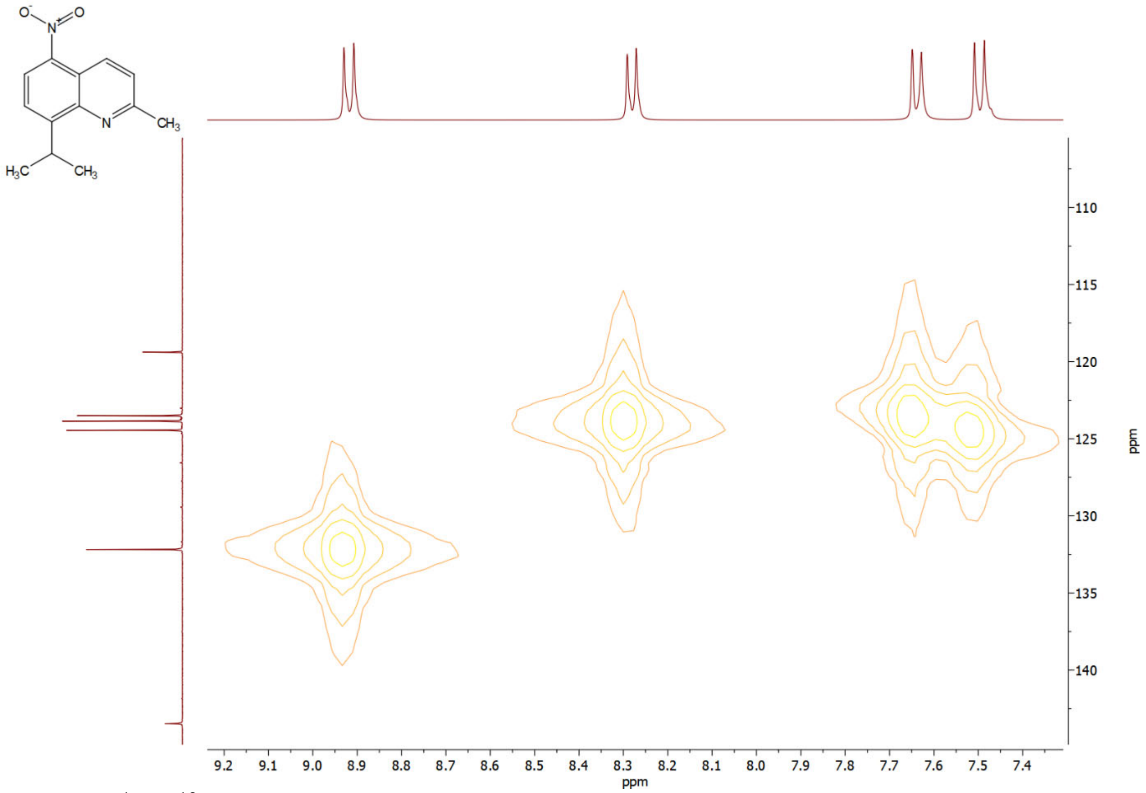


**Fig. S4d.** $^{1}H$ - $^{13}C$ HMQC spectrum (aromatic range) of the **4d**.

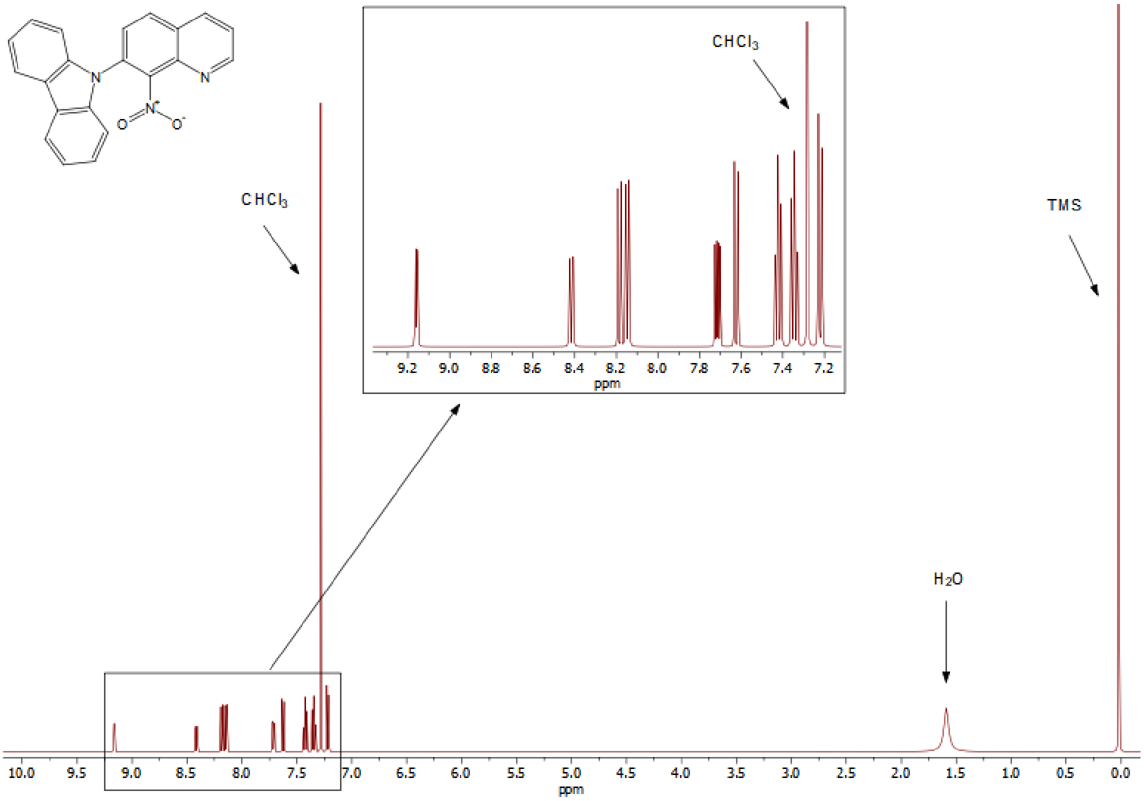


**Fig. S5a.** $^{1}H$ NMR ($CDCl_3$; 500.2 MHz) spectrum of the **5a**.

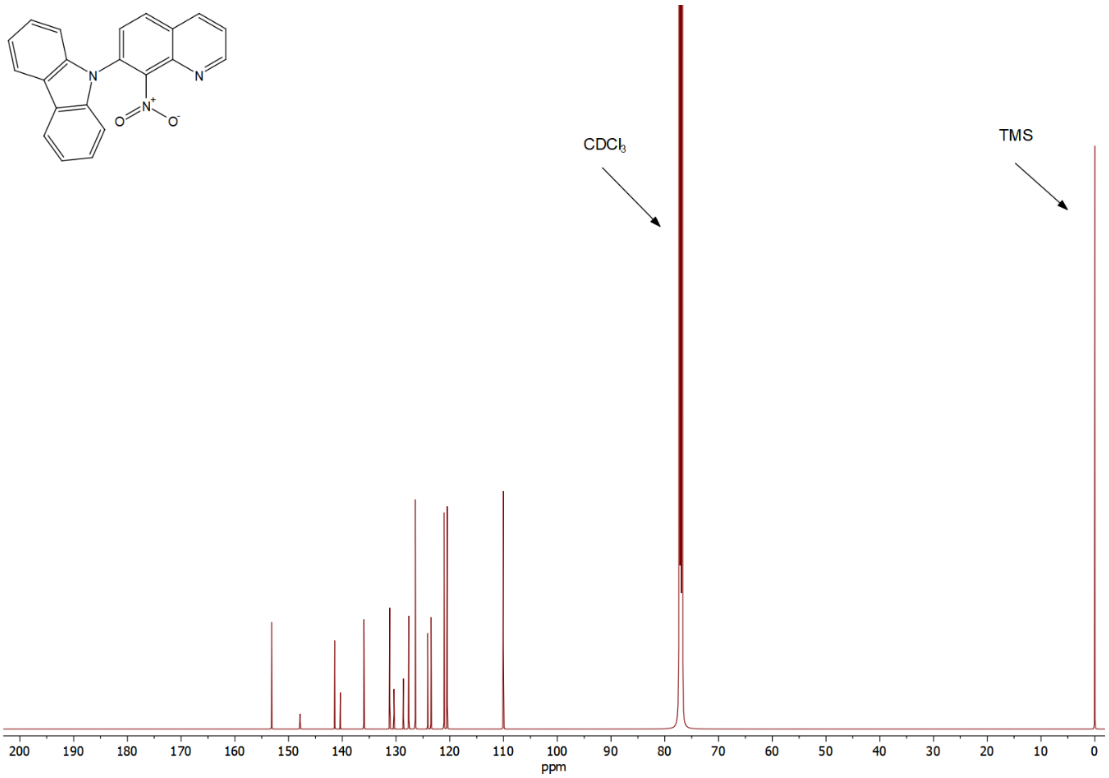


**Fig. S5b.** $^{13}C\{^{1}H\}$ NMR ($CDCl_3$; 125.8 MHz) spectrum of the **5a**.

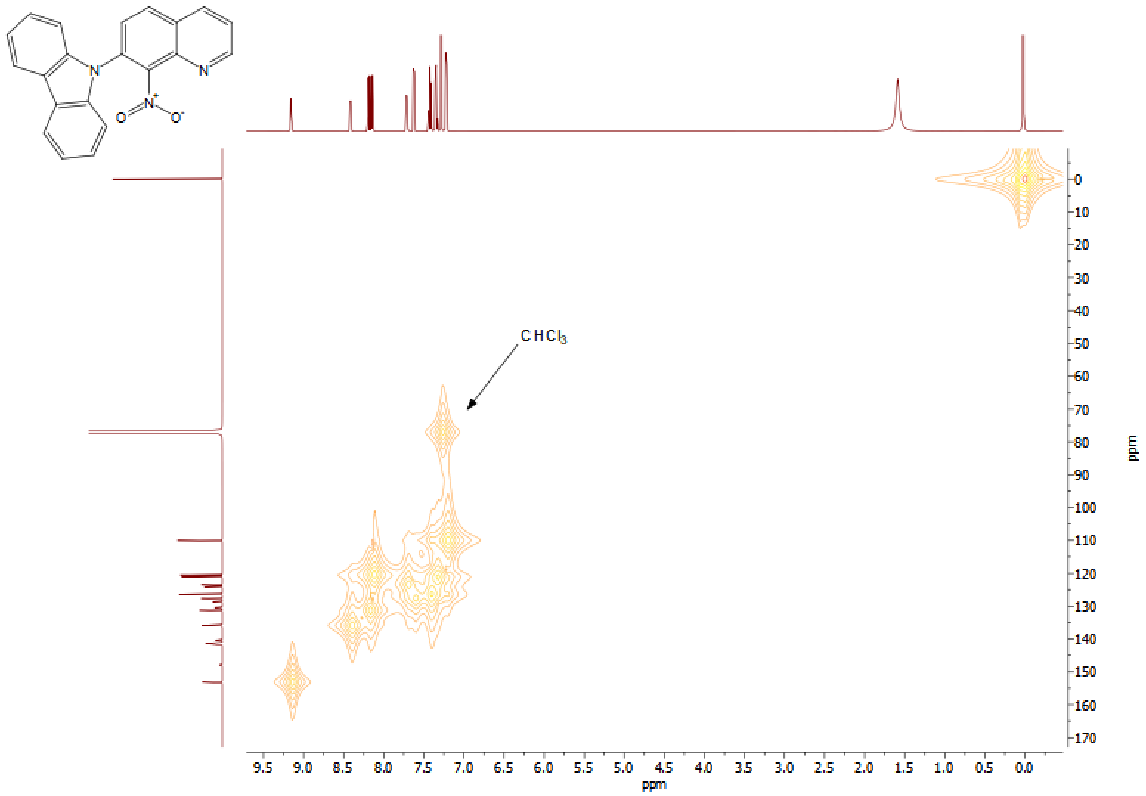


**Fig. S5c.** $^{1}H$ - $^{13}C$ HMQC spectrum of the **5a**.

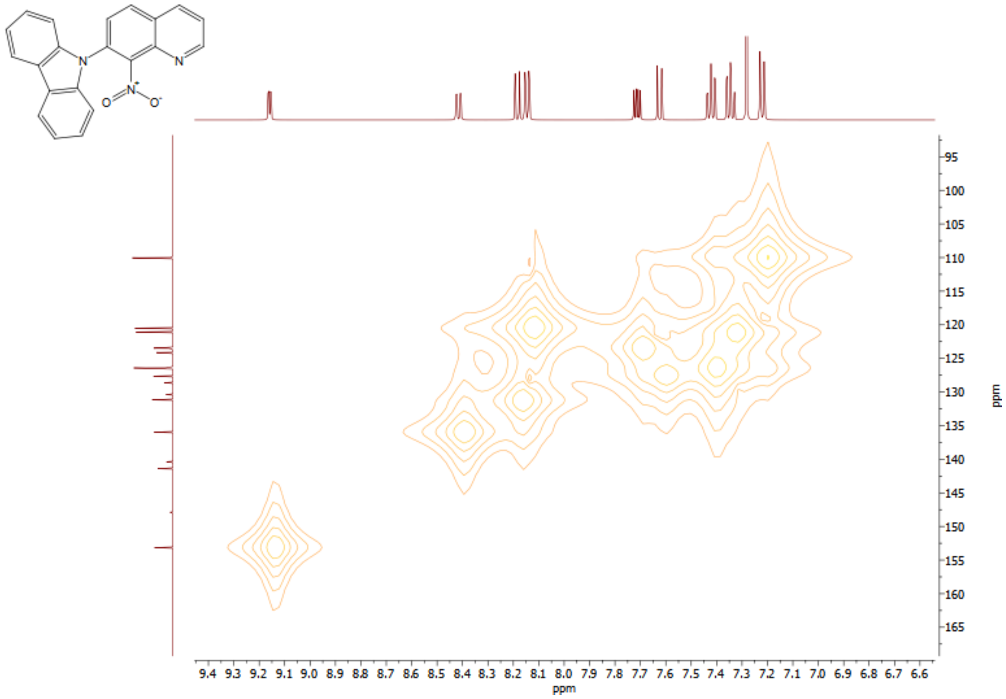


**Fig. S5d.** $^{1}H$ - $^{13}C$ HMQC spectrum (aromatic range) of the **5a**.

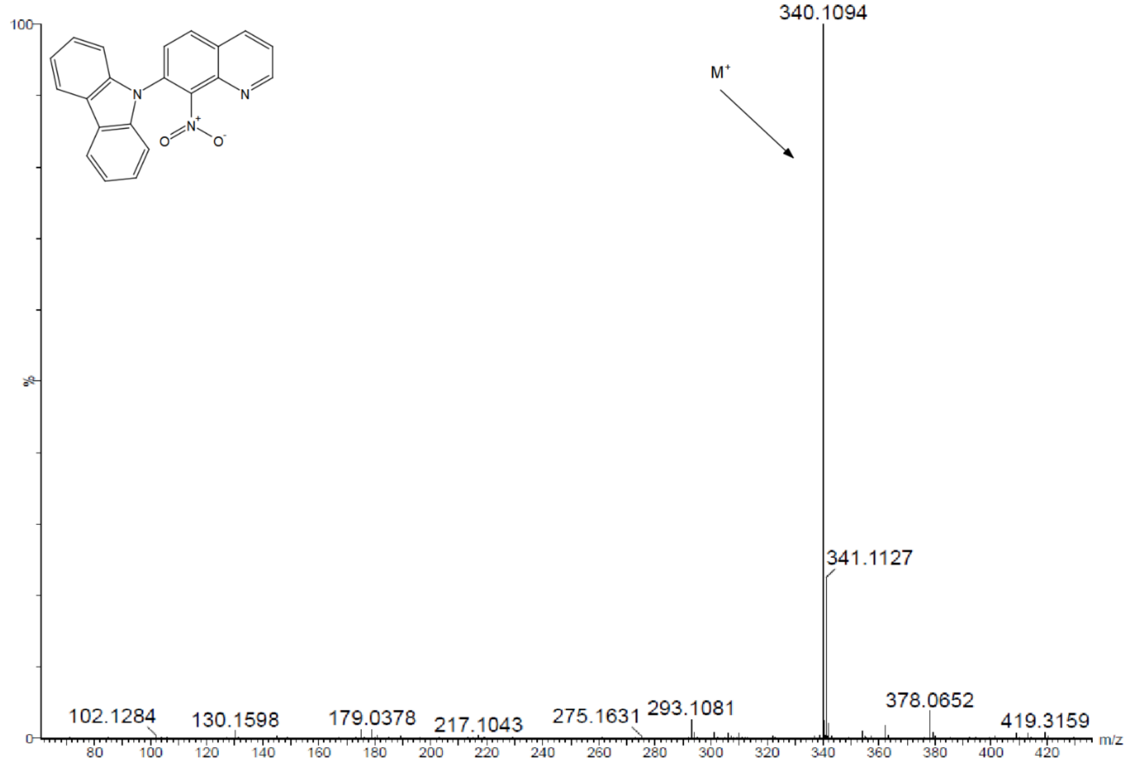


**Fig. S5e.** MS spectrum of the **5a**.

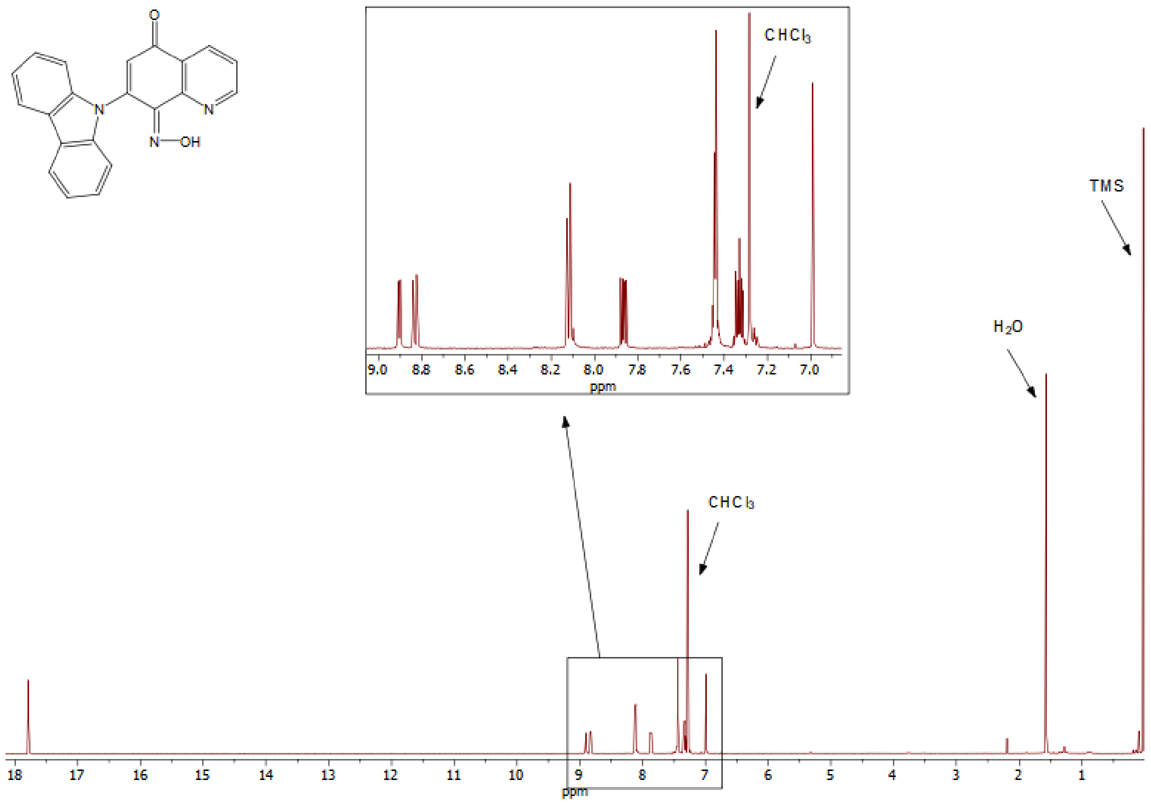


**Fig. S6a.** $^1$H NMR ($CDCl_3$; 500.2 MHz) spectrum of the **5b**.

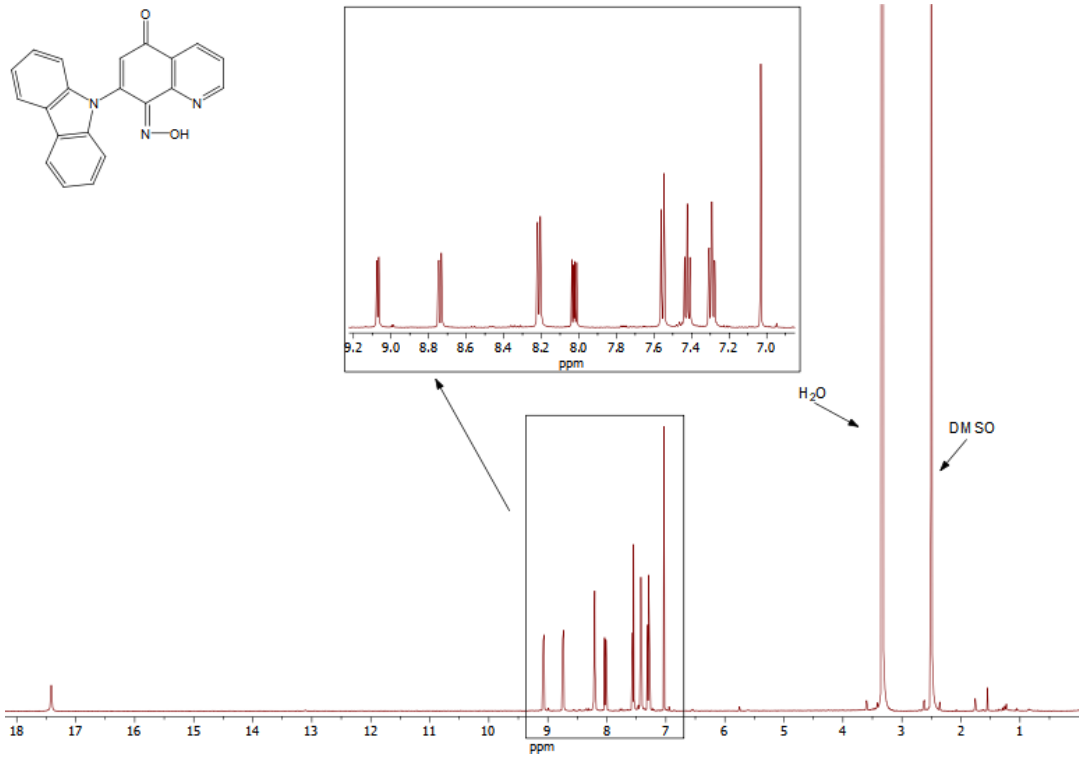


**Fig. S6b.** $^1$H NMR (DMSO-$d_6$; 500.2 MHz) spectrum of the **5b**.

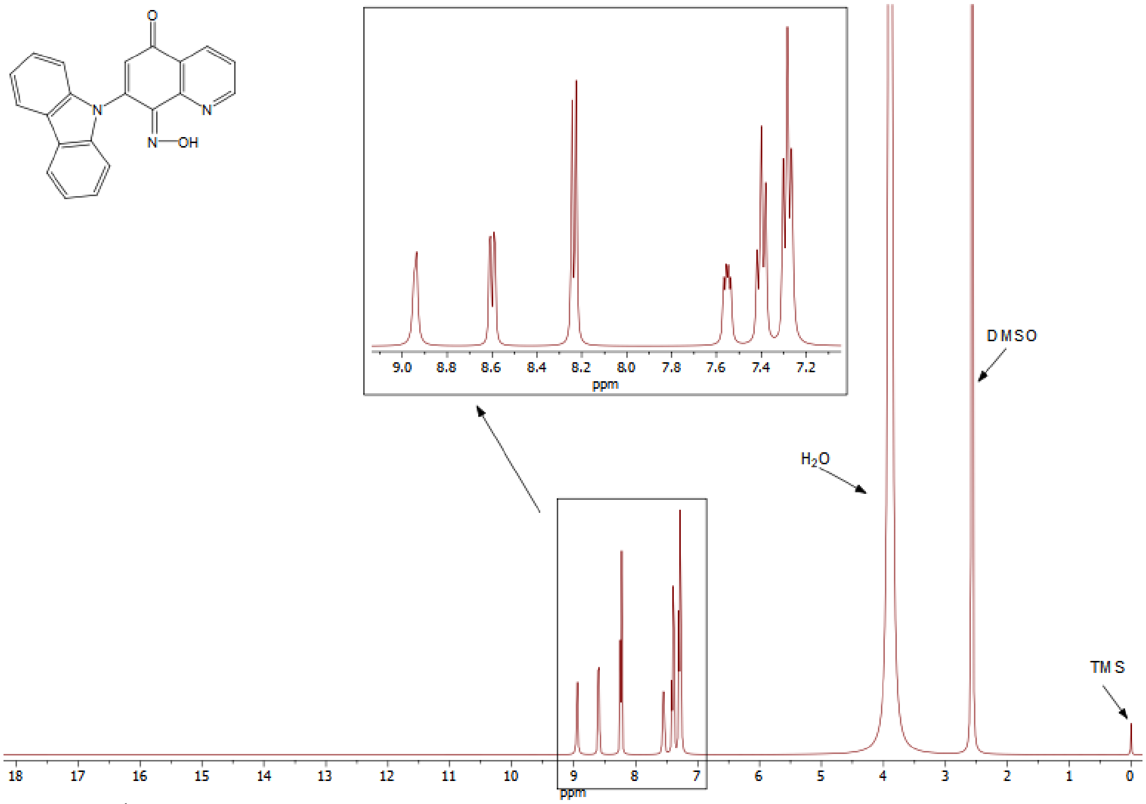


**Fig. S6c.** $^{1}$H NMR (DMSO–d6/KOD/$D_2O$; 500.2 MHz) spectrum of the **5b**

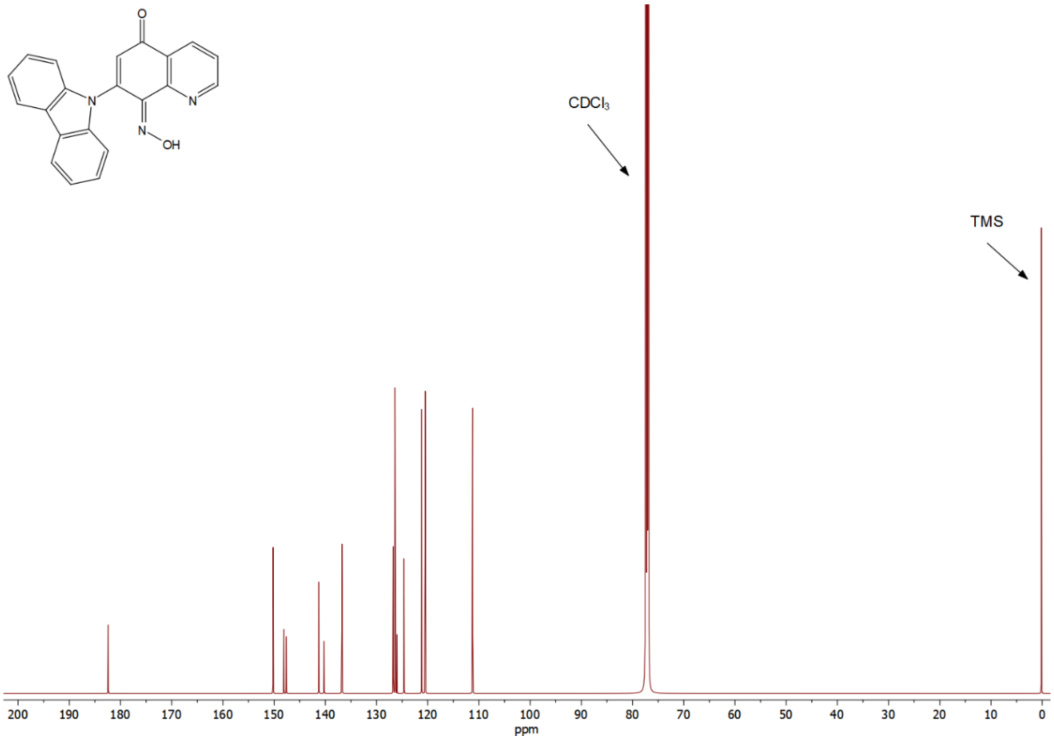


**Fig. S6d.** $^{13}$C{$^{1}$H} NMR ($CDCl_3$; 125.8 MHz) spectrum of the **5b**.

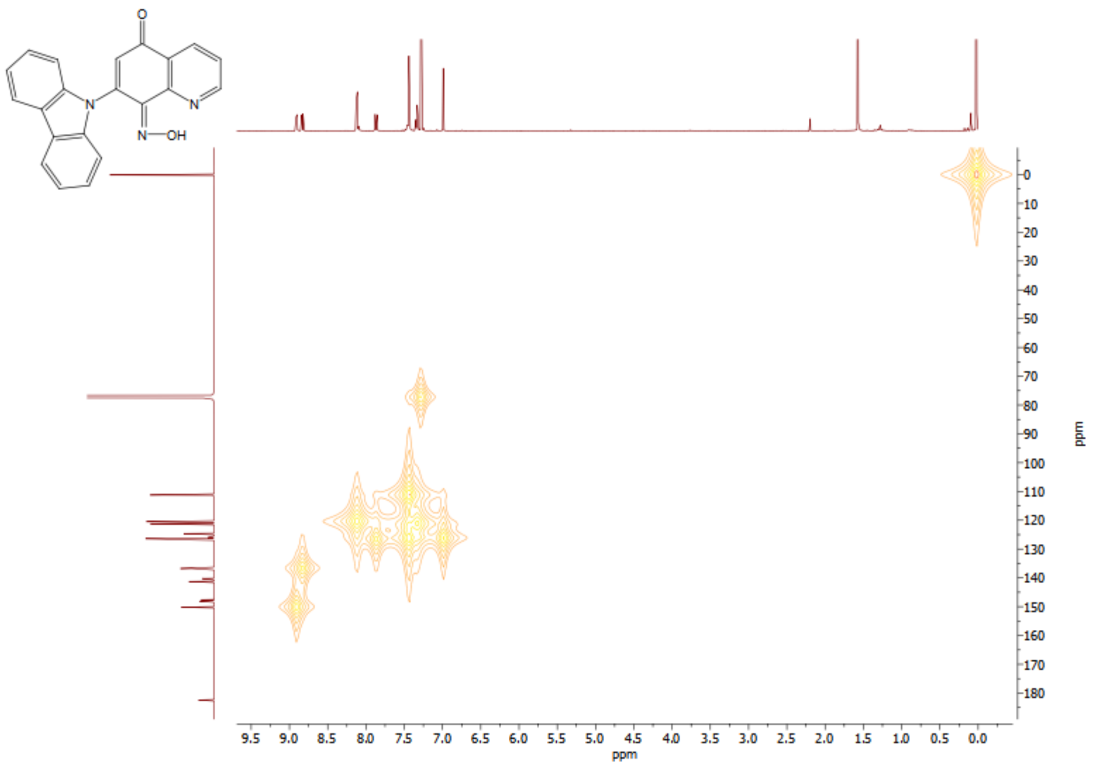


**Fig. S6e.** $^{1}H$ - $^{13}C$ HMQC spectrum of the **5b**.

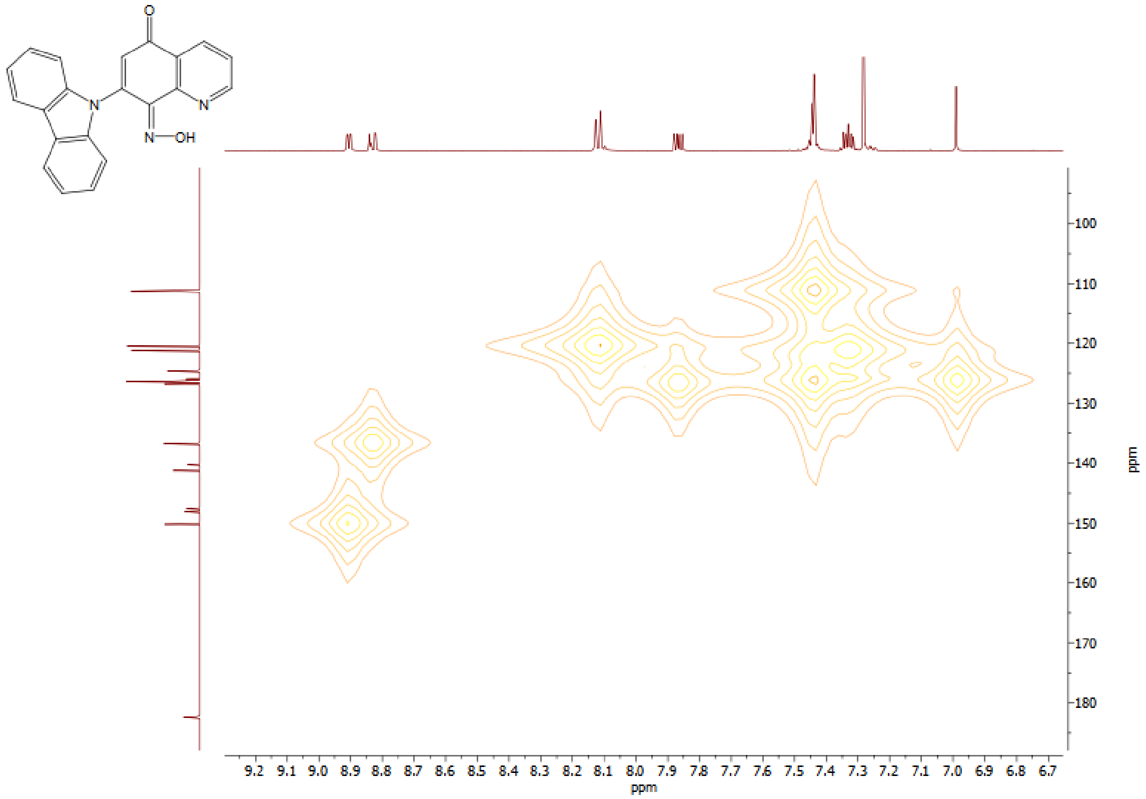


**Fig. S6f.** $^{1}H$ - $^{13}C$ HMQC spectrum (aromatic range) of the **5b**.

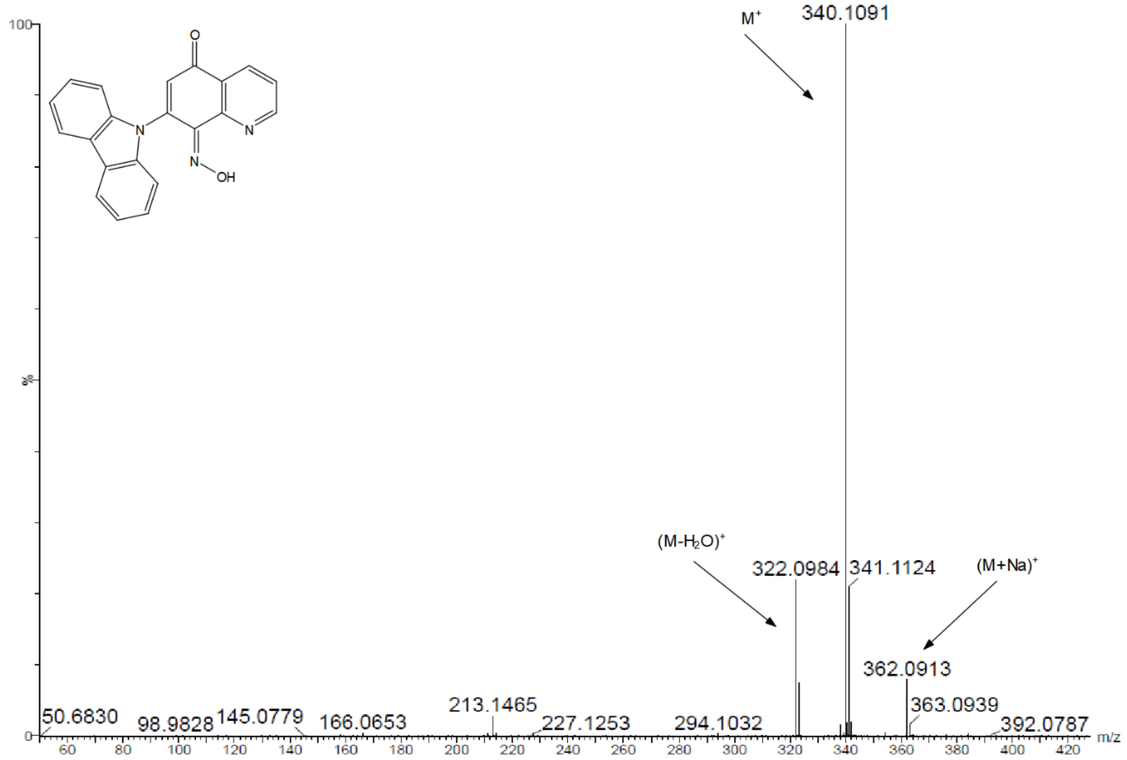


**Fig. S6g.** MS spectrum of the **5b**.

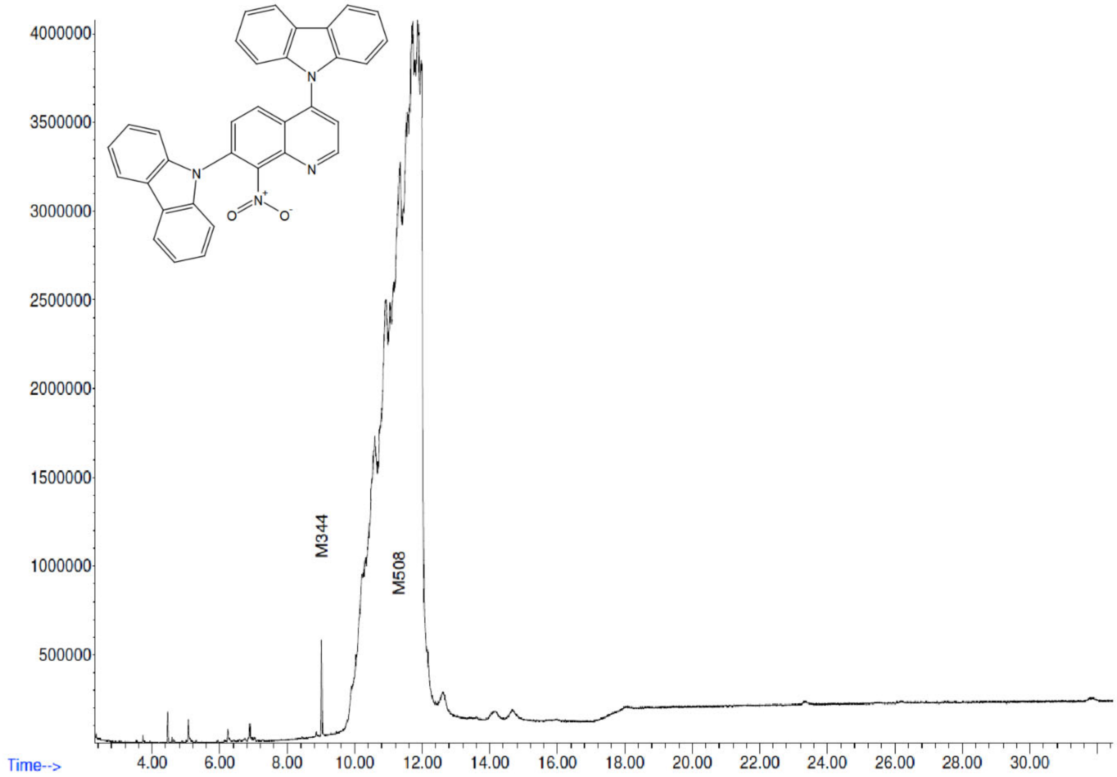


**Fig. S7a.** Chromatogram of the structure similar to molecule **5c**.

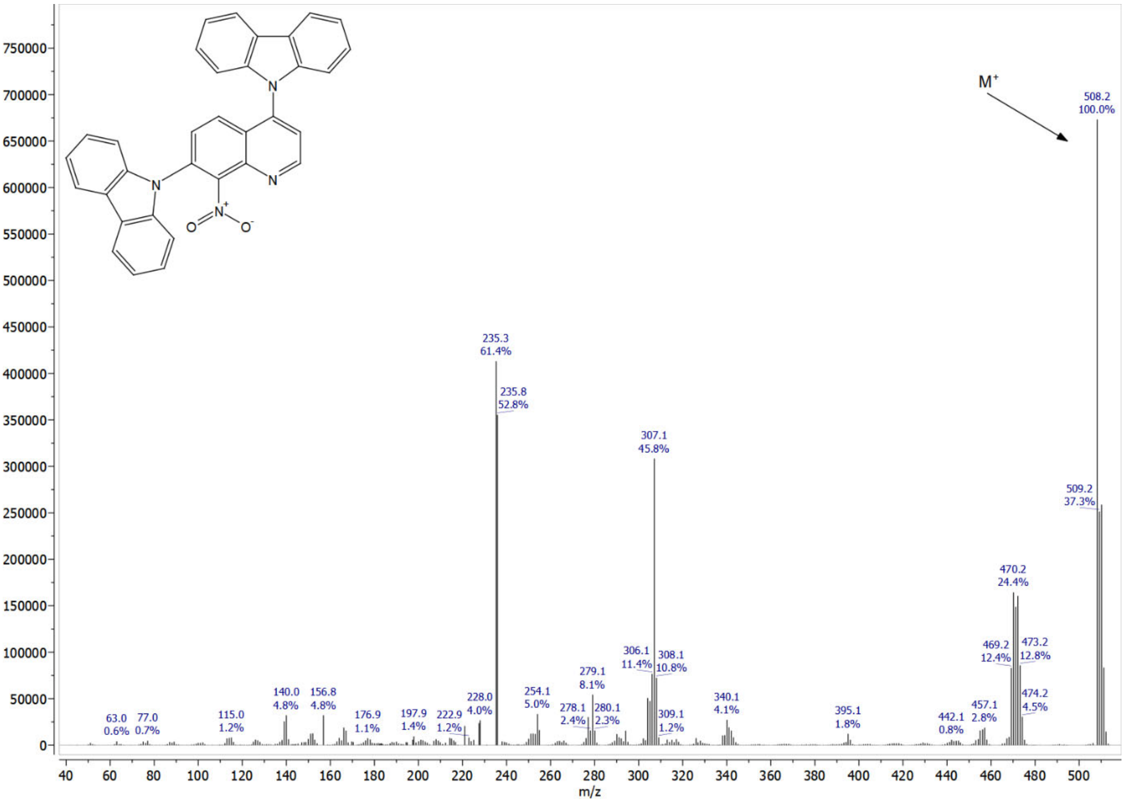


**Fig. S7b.** MS of the structure similar to molecule **5c**.

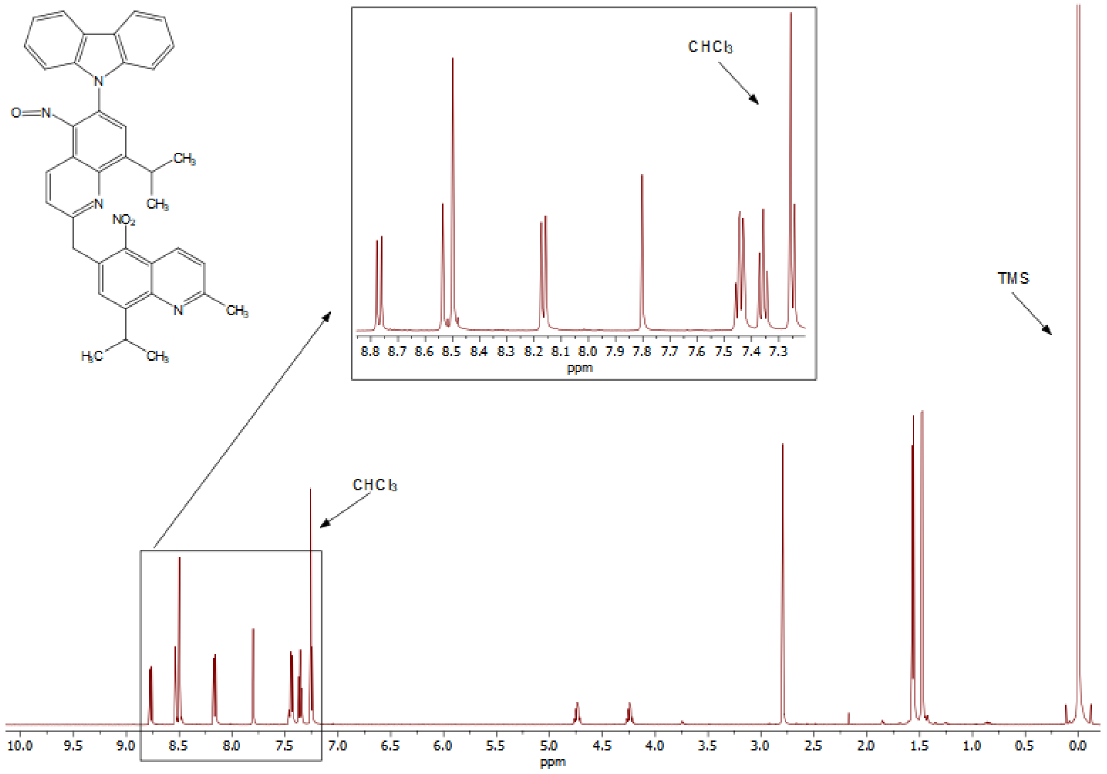


**Fig. S8a.** $^{1}$H NMR ($CDCl_3$; 500.2 MHz) spectrum of the **5d**.

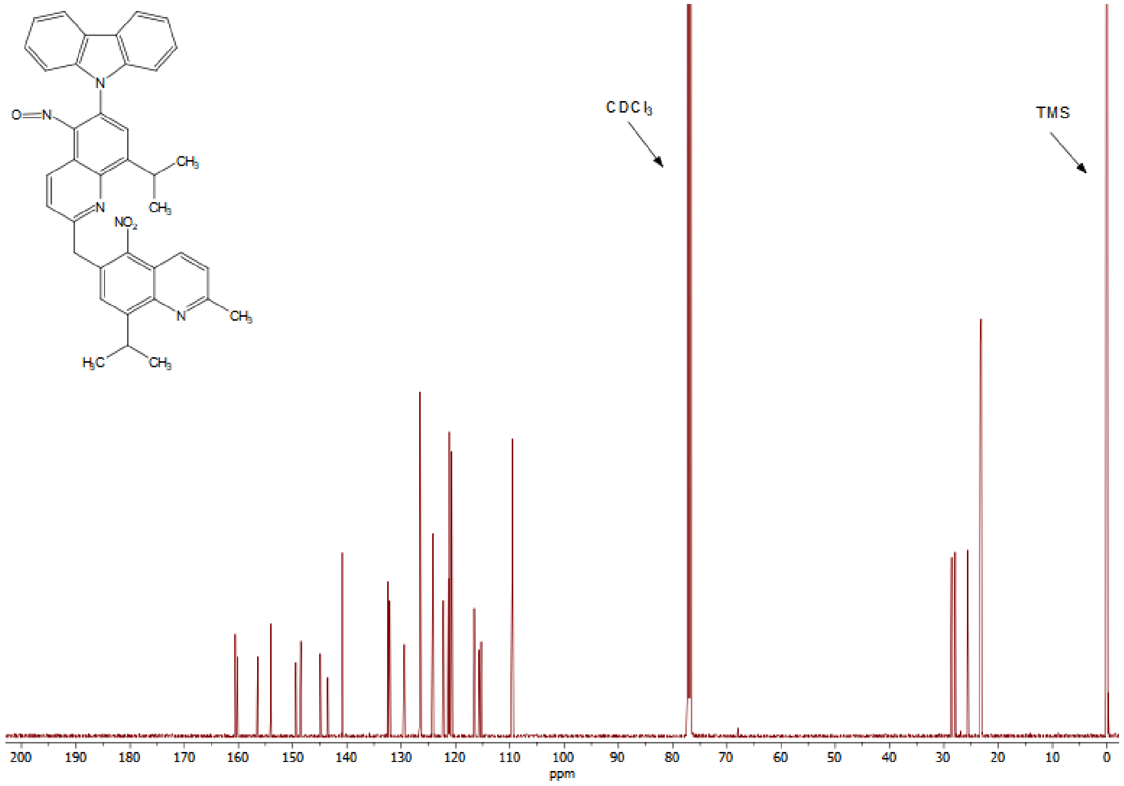


**Fig. S8b.** $^{13}C\{^{1}H\}$ NMR ($CDCl_3$; 125.8 MHz) spectrum of the **5d**.

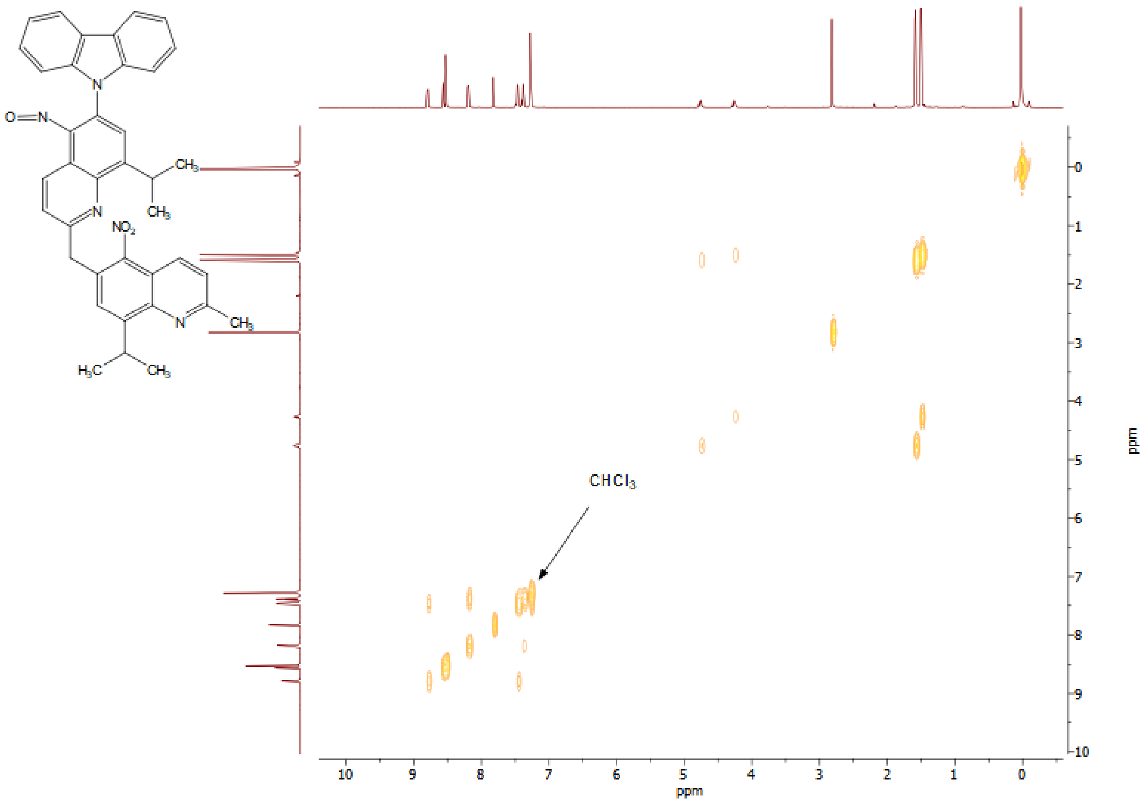


**Fig. S8c.** $^1$H – $^1$H COSY spectrum of the **5d**.

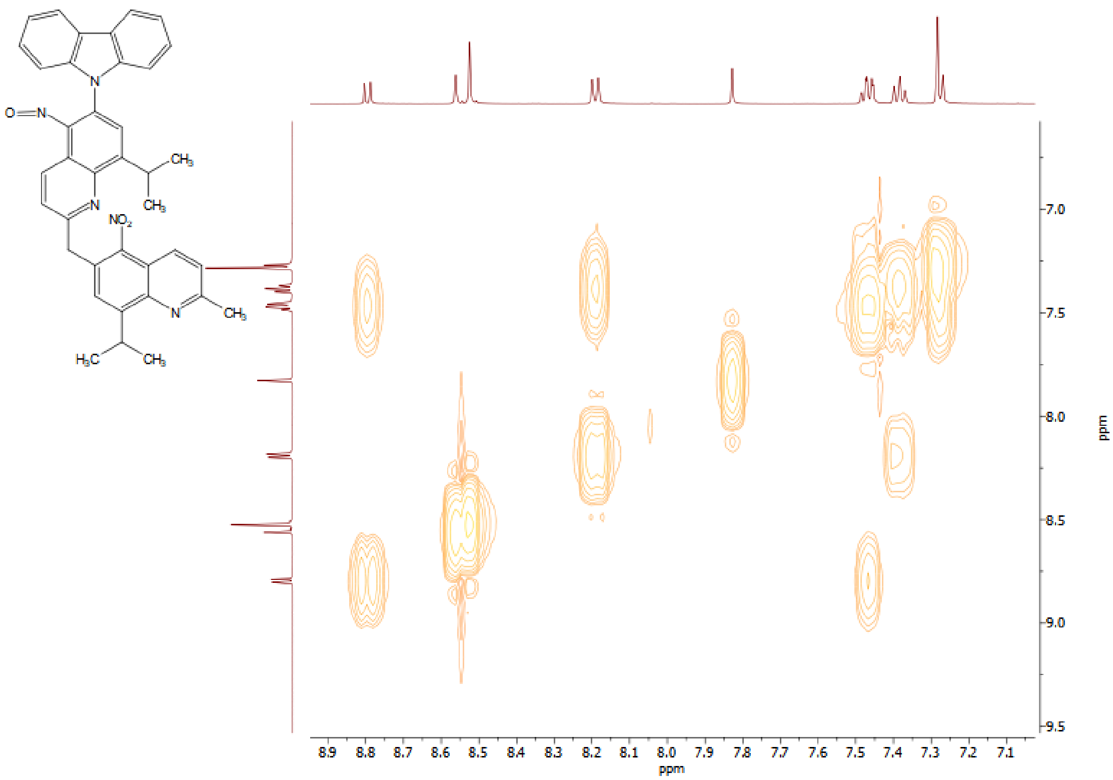


**Fig. S8d.** $^1$H – $^1$H COSY spectrum (aromatic range) of the **5d**.

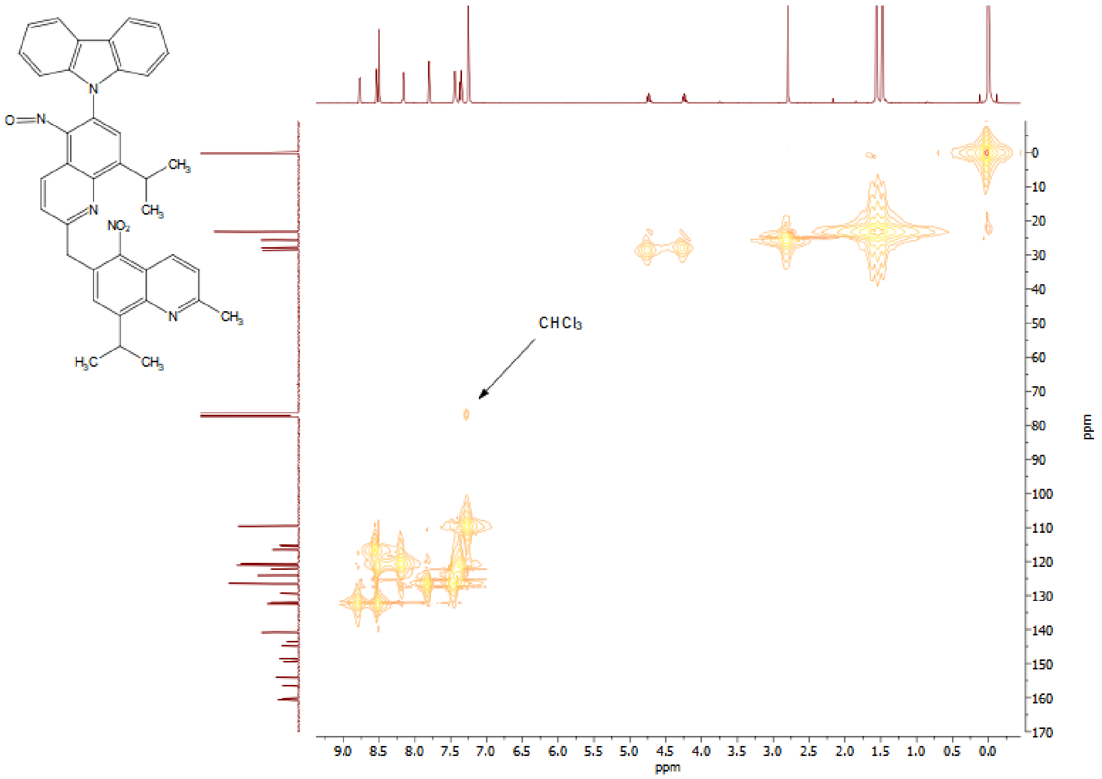


**Fig. S8e.** $^{1}H$ - $^{13}C$ HMQC spectrum of the **5d**.

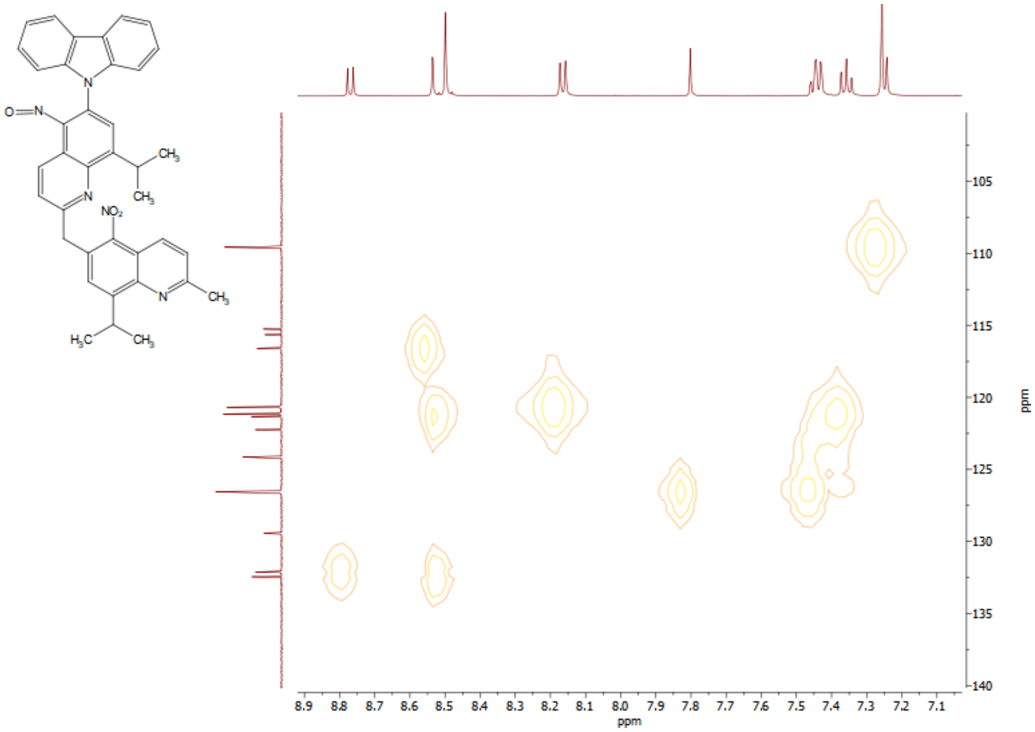


**Fig. S8f.** $^{1}H$ - $^{13}C$ HMQC spectrum (aromatic range) of the **5d**.

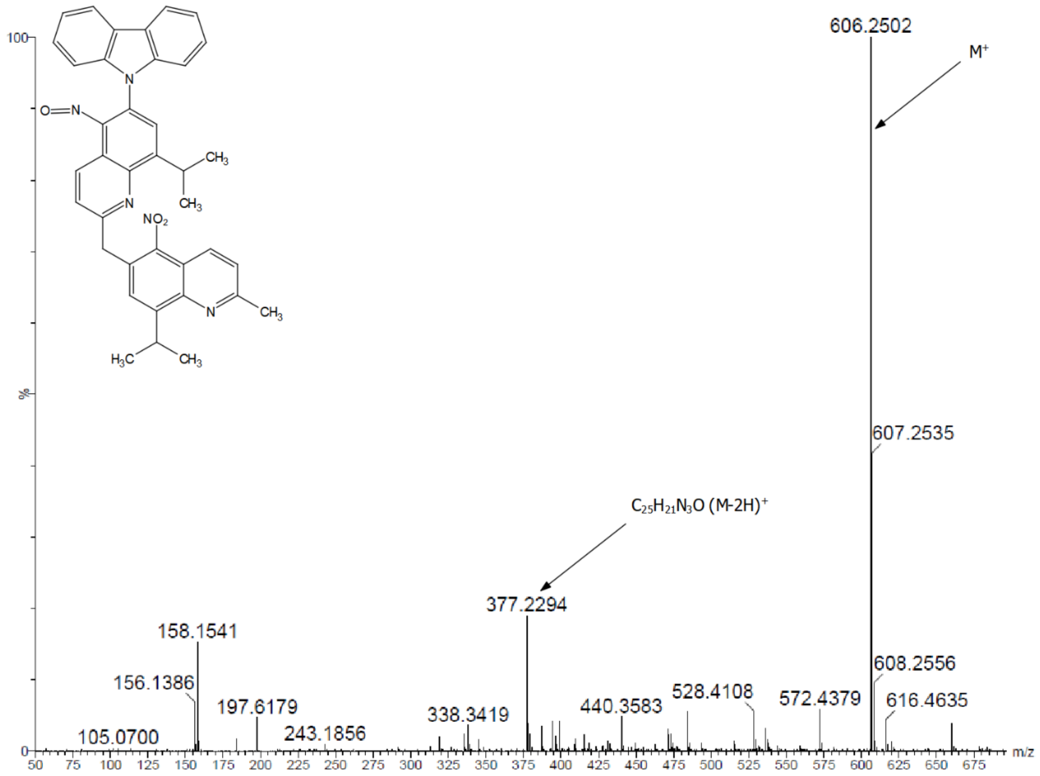


**Fig. S8g.** MS (ES-TOF) spectrum of the **5d**.

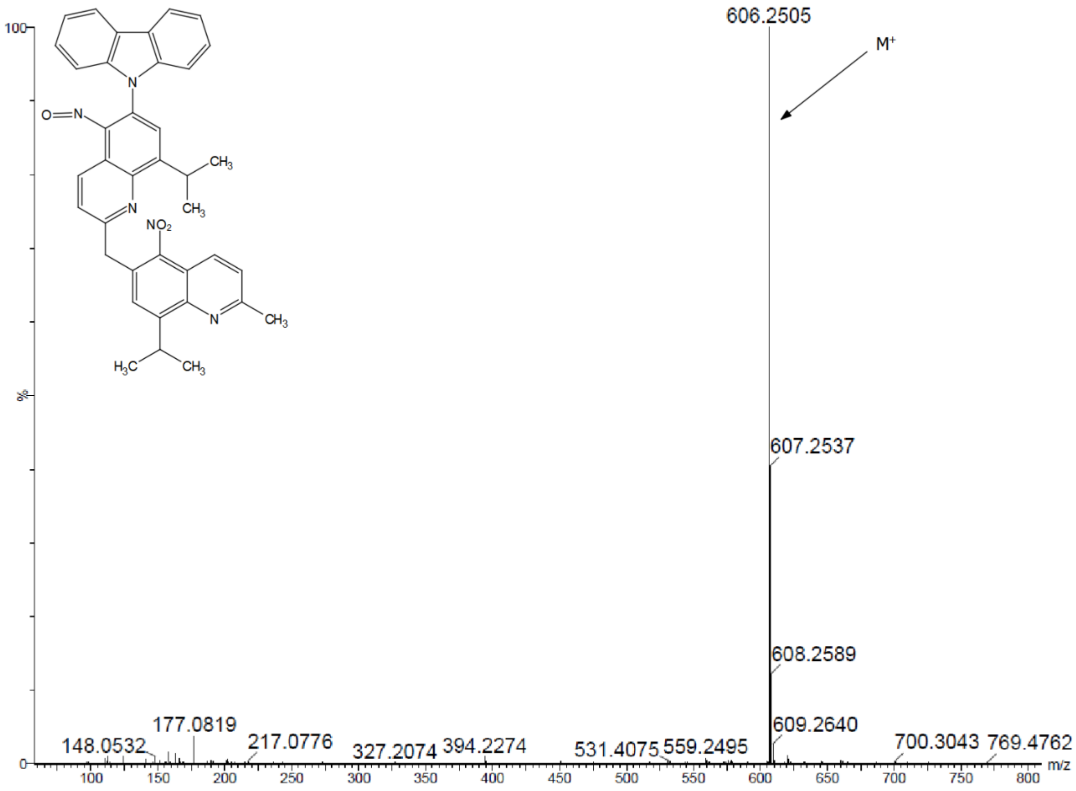


**Fig. S8e.** MS (AP-TOF) spectrum of the **5d**.

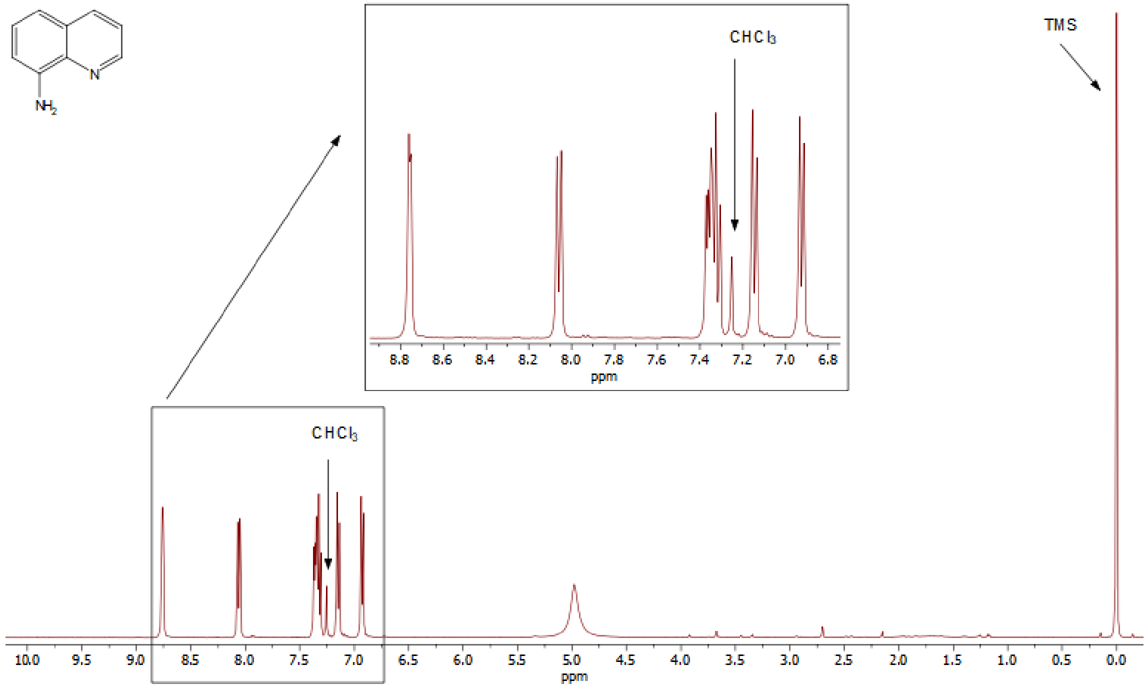


**Fig. S9.** $^1$H NMR ($CDCl_3$; 400.2 MHz) spectrum of the **6a**.

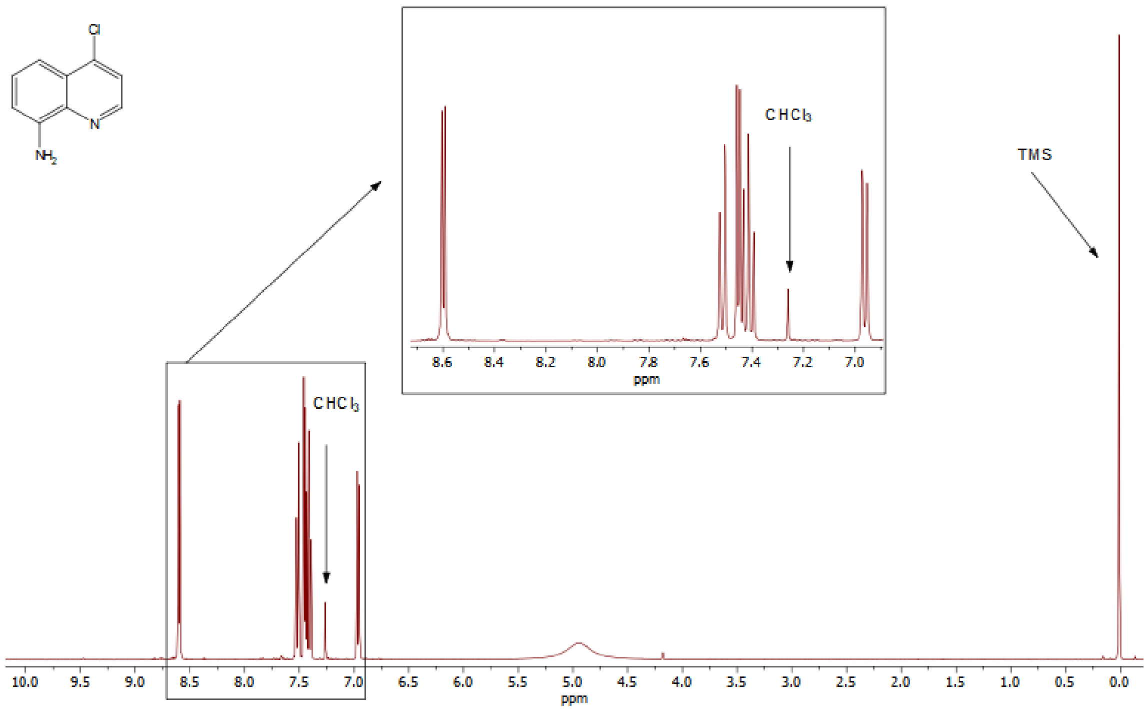


**Fig. S10a.** $^{1}$H NMR ($CDCl_3$; 400.2 MHz) spectrum of the **6b**.

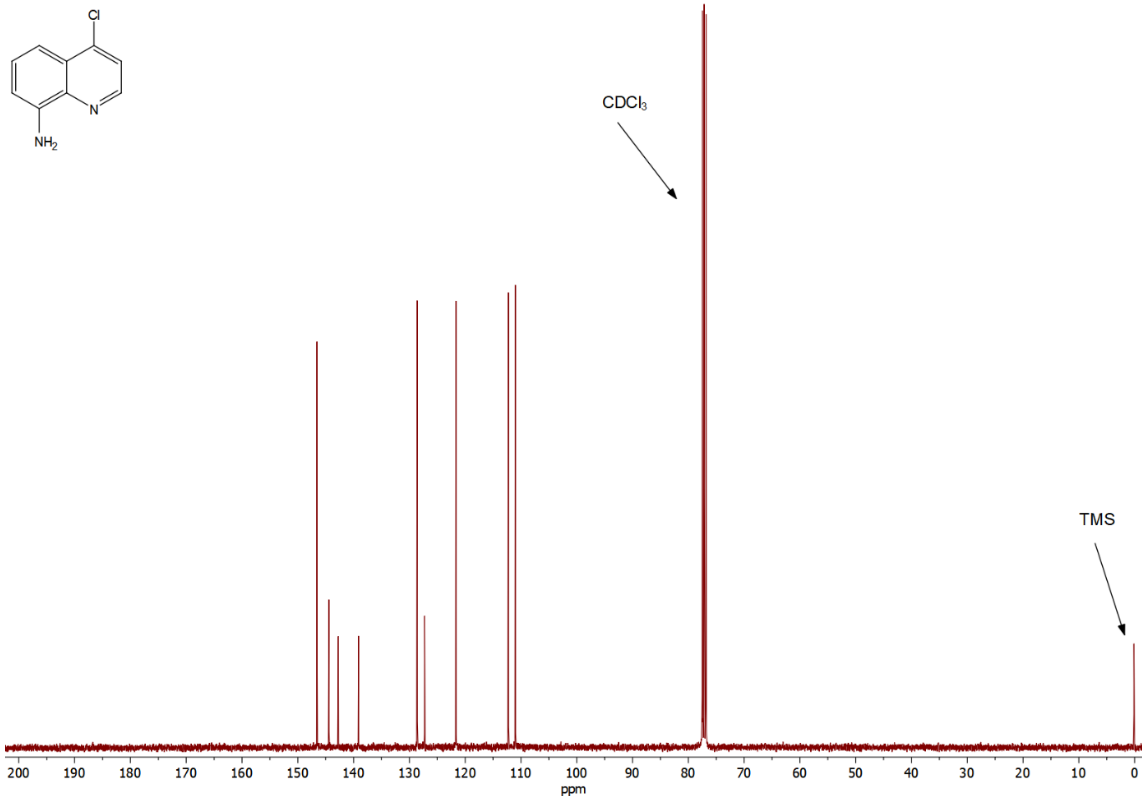


**Fig. S10b.** $^{13}C\{^{1}H\}$ NMR ($CDCl_3$; 100.6 MHz) spectrum of the **6b**.

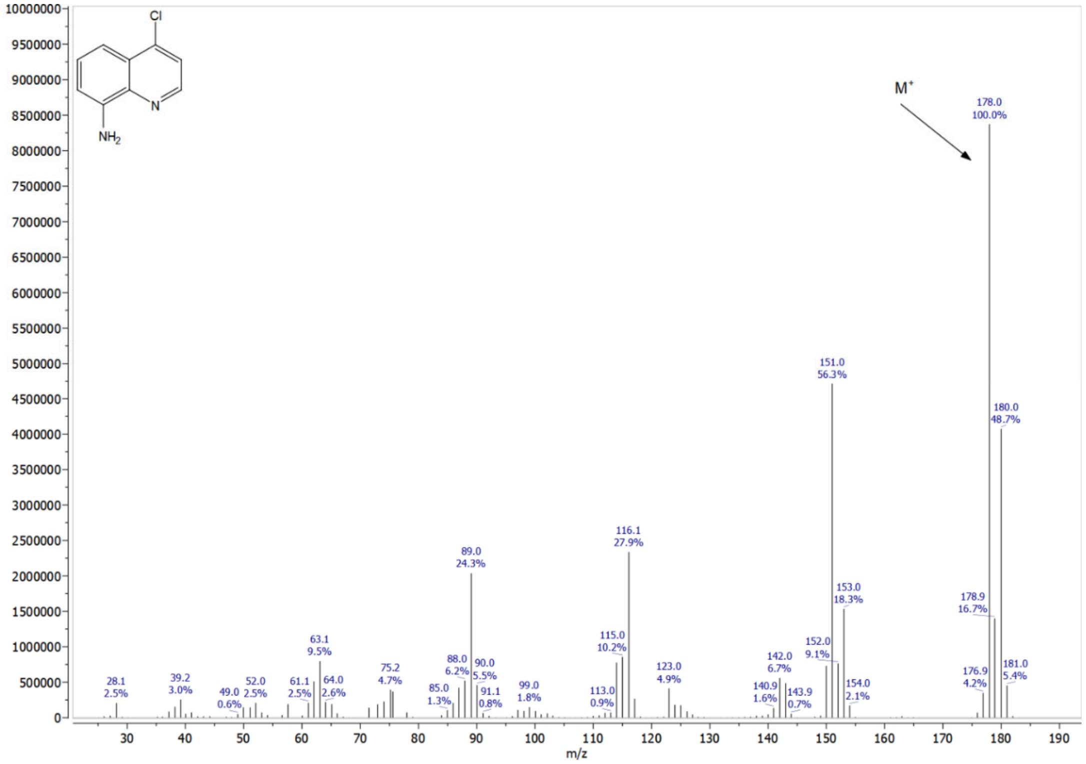


**Fig. S10c.** MS spectrum of the **6b**.

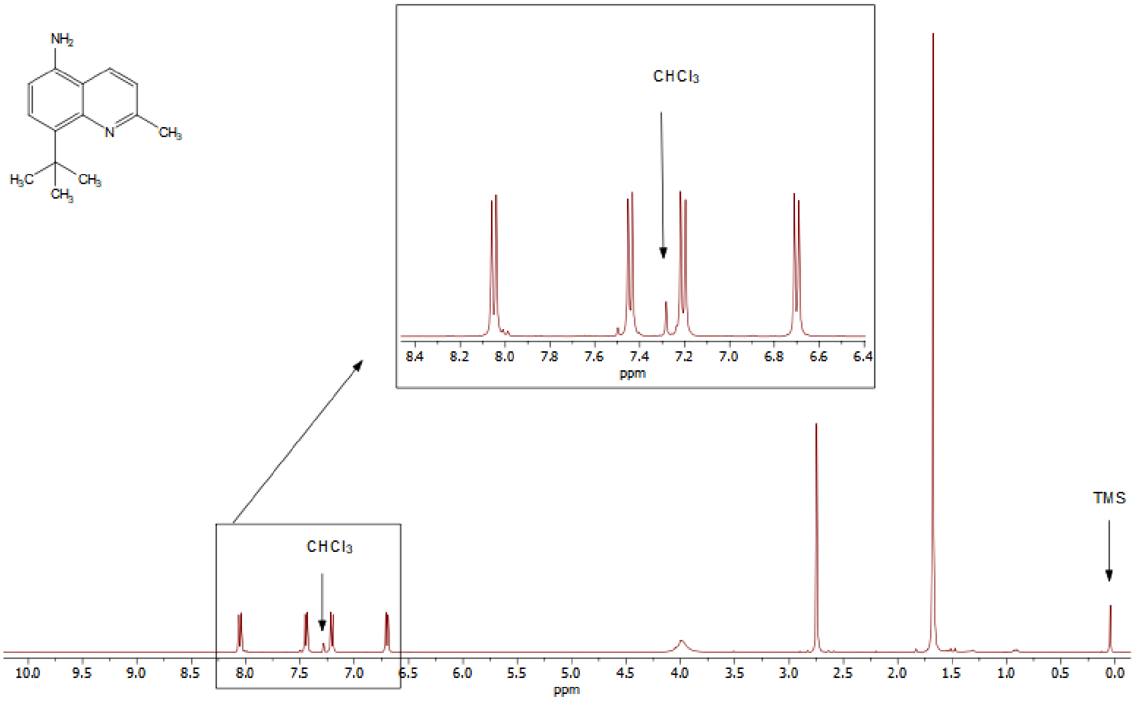


**Fig. S11a.** $^{1}H$ NMR ($CDCl_3$; 400.2 MHz) spectrum of the **6c**.

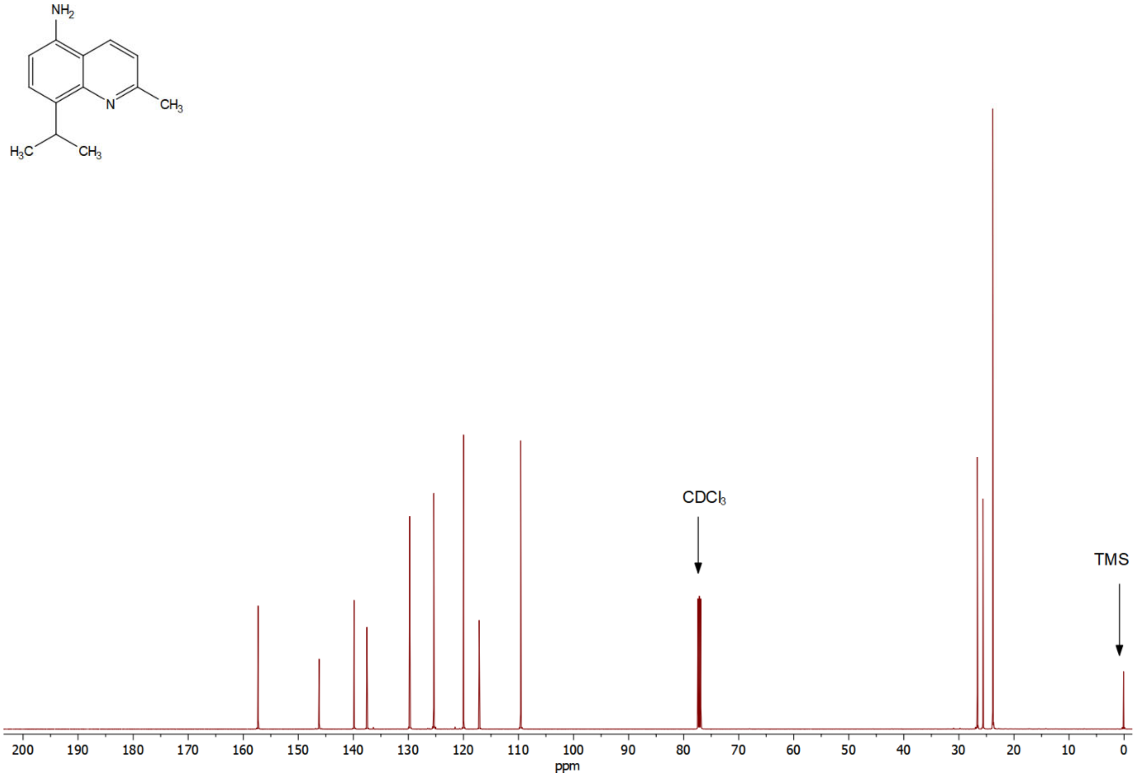


**Fig. S11b.** $^{13}C\{^{1}H\}$ NMR ($CDCl_3$; 100.6 MHz) spectrum of the **6c**.

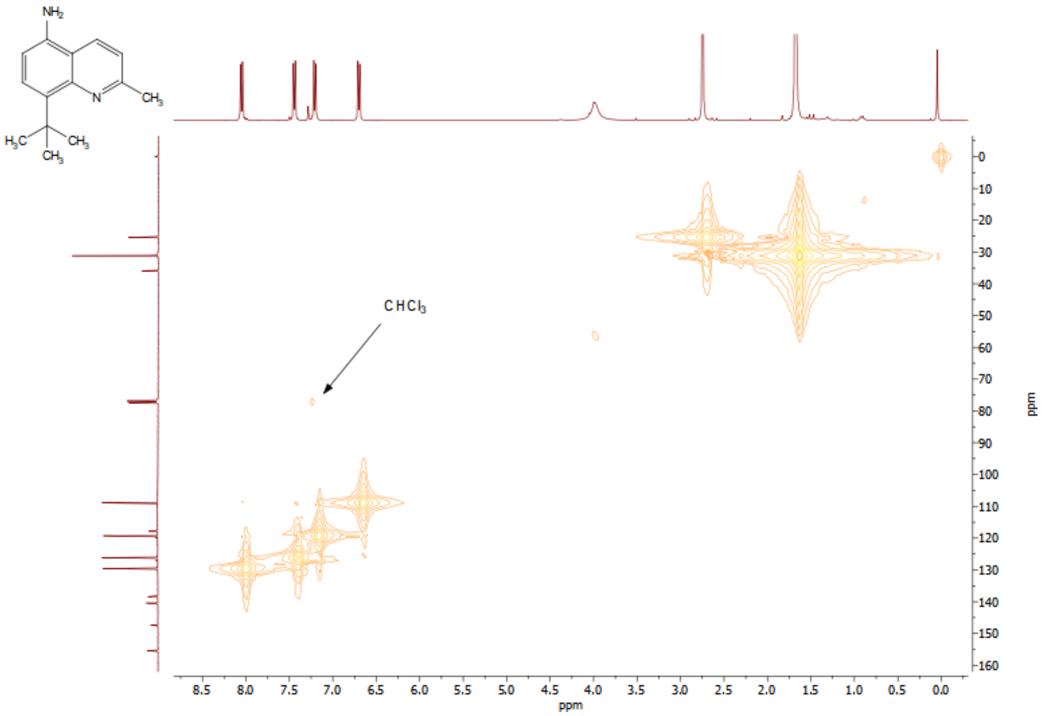


**Fig. S11c.** $^{1}H$ - $^{13}C$ HMQC spectrum of the **6c**.

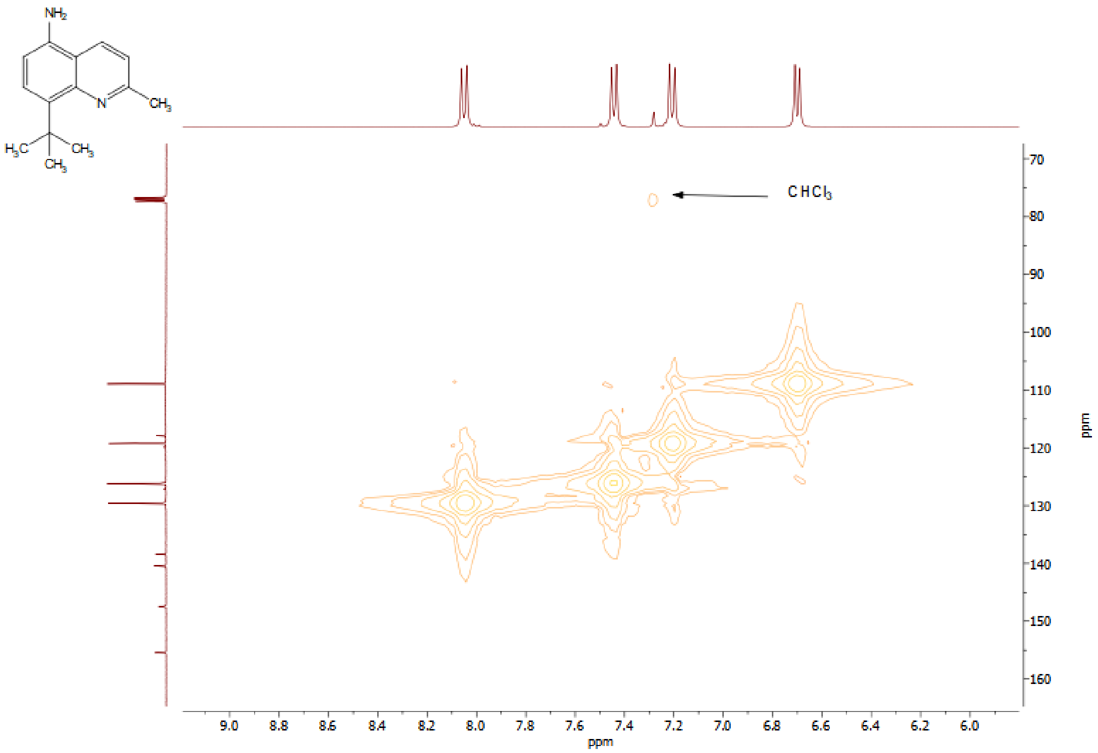


**Fig. S11d.** $^{1}H$ - $^{13}C$ HMQC spectrum (aromatic range) of the **6c**.

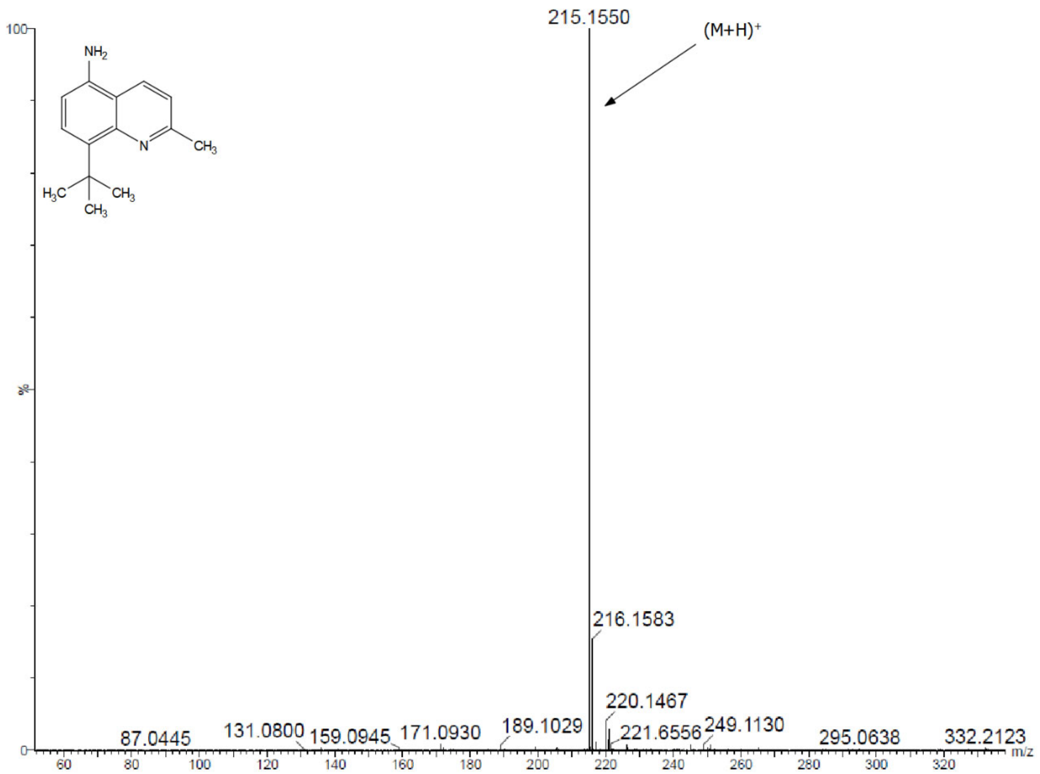


**Fig. S11e.** MS spectrum of the **6c**.

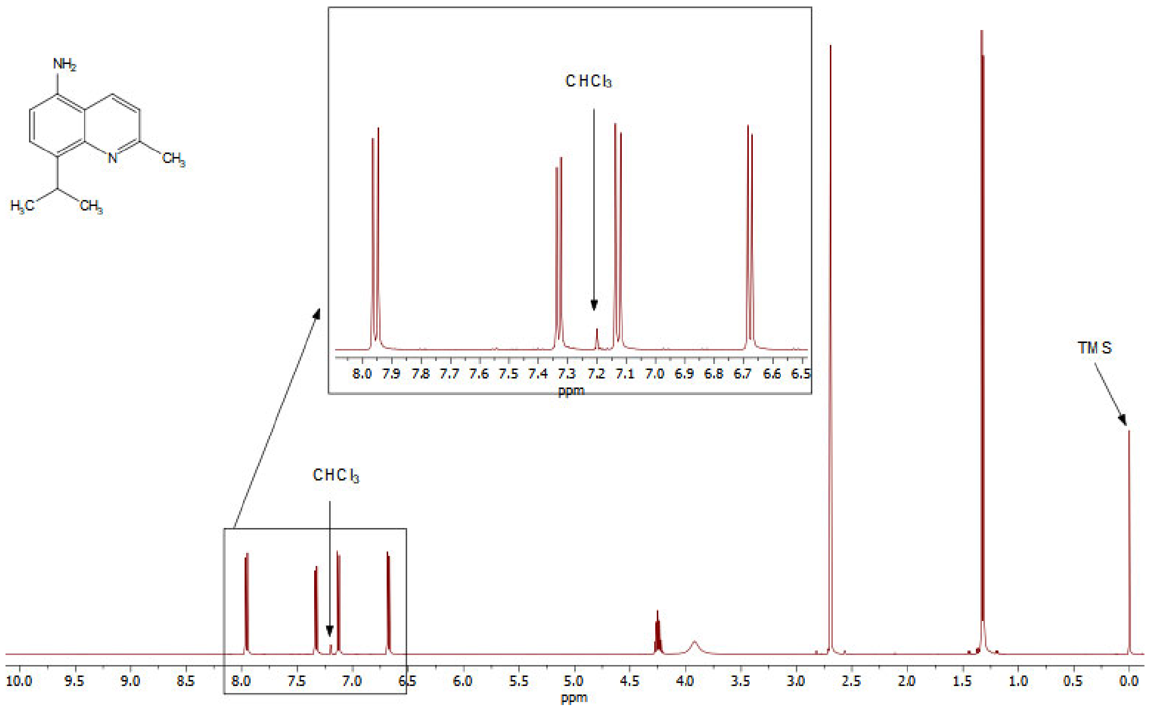


**Fig. S12a.** $^{1}H$ NMR ($CDCl_3$; 500.2 MHz) spectrum of the **6d**.

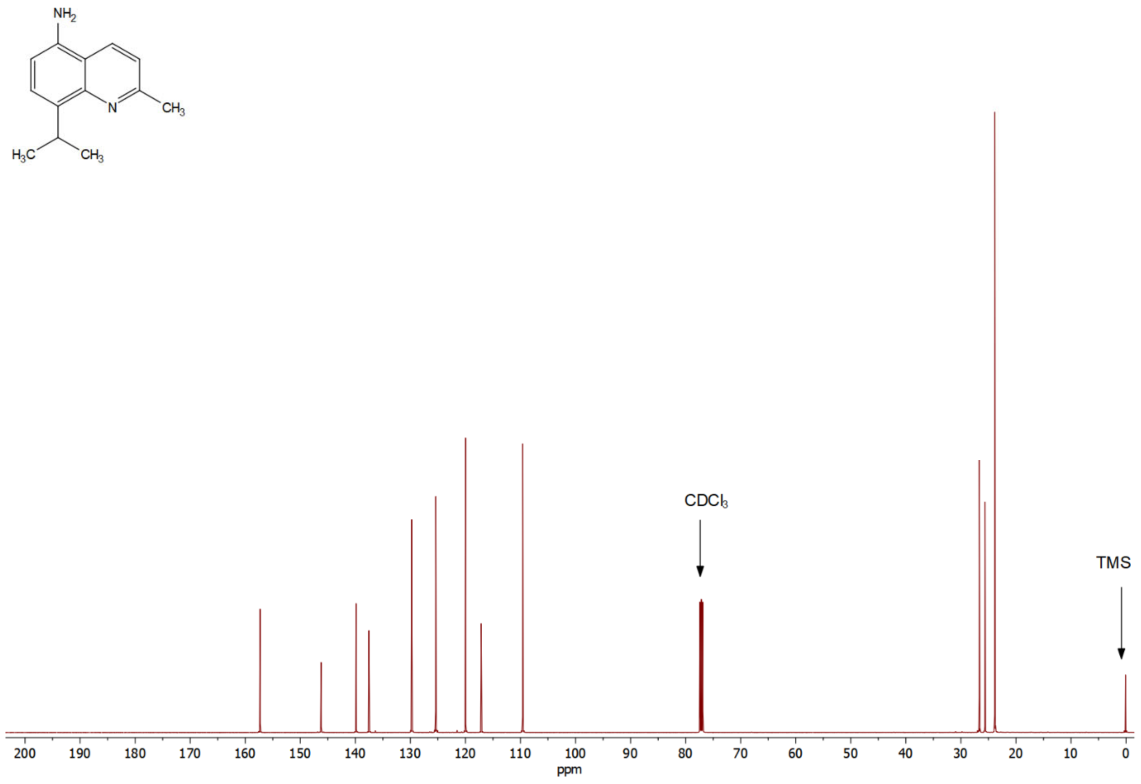


**Fig. S12b.** $^{13}C\{^{1}H\}$ NMR ($CDCl_3$; 125.8 MHz) spectrum of the **6d**.

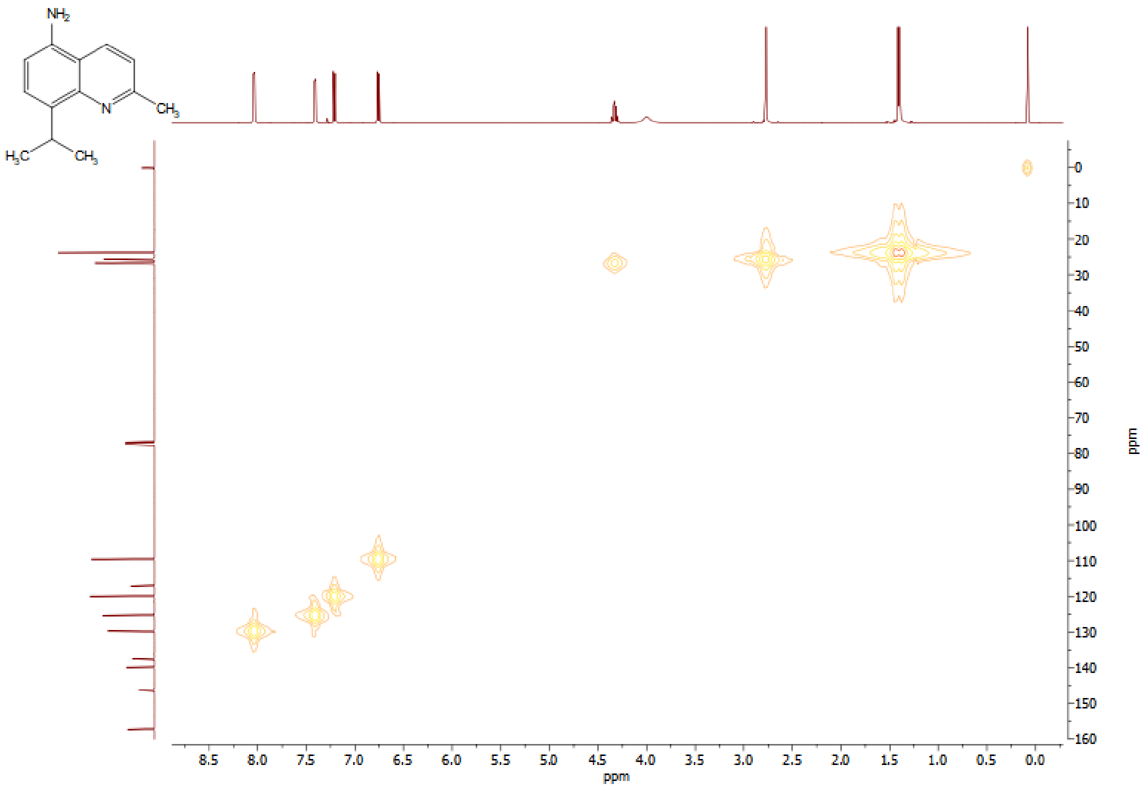


**Fig. S12c.** $^{1}H$ - $^{13}C$ HMQC spectrum of the **6d**.

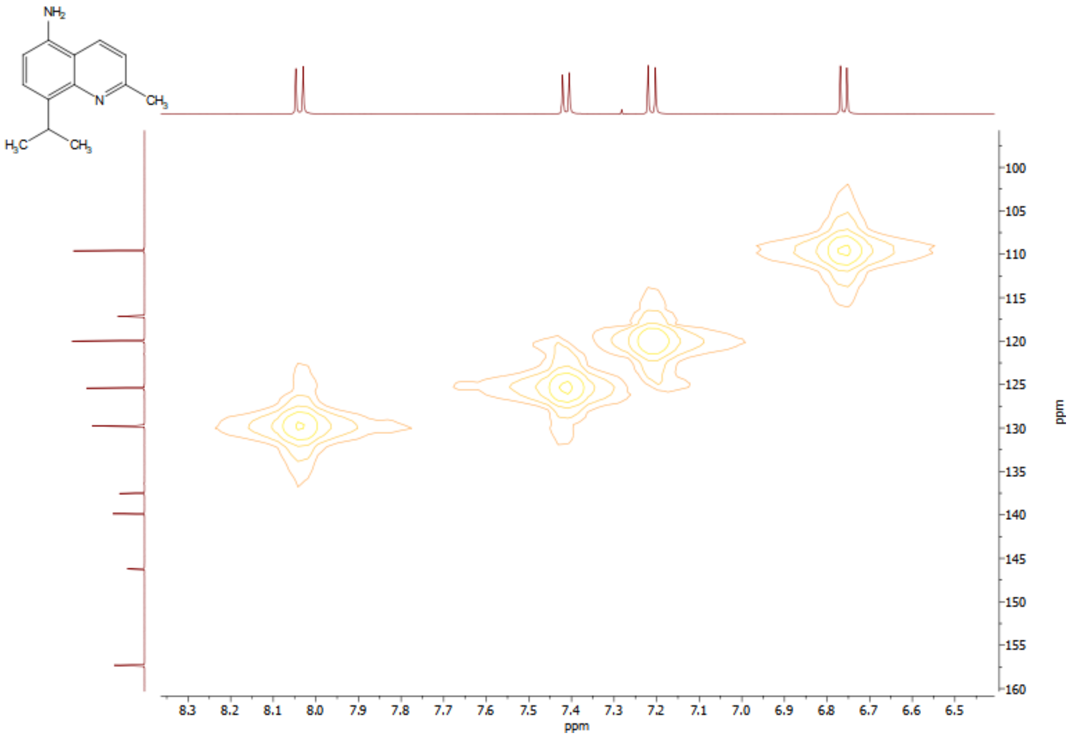


**Fig. S12d.** $^{1}H$ - $^{13}C$ HMQC spectrum (aromatic range) of the **6d**.

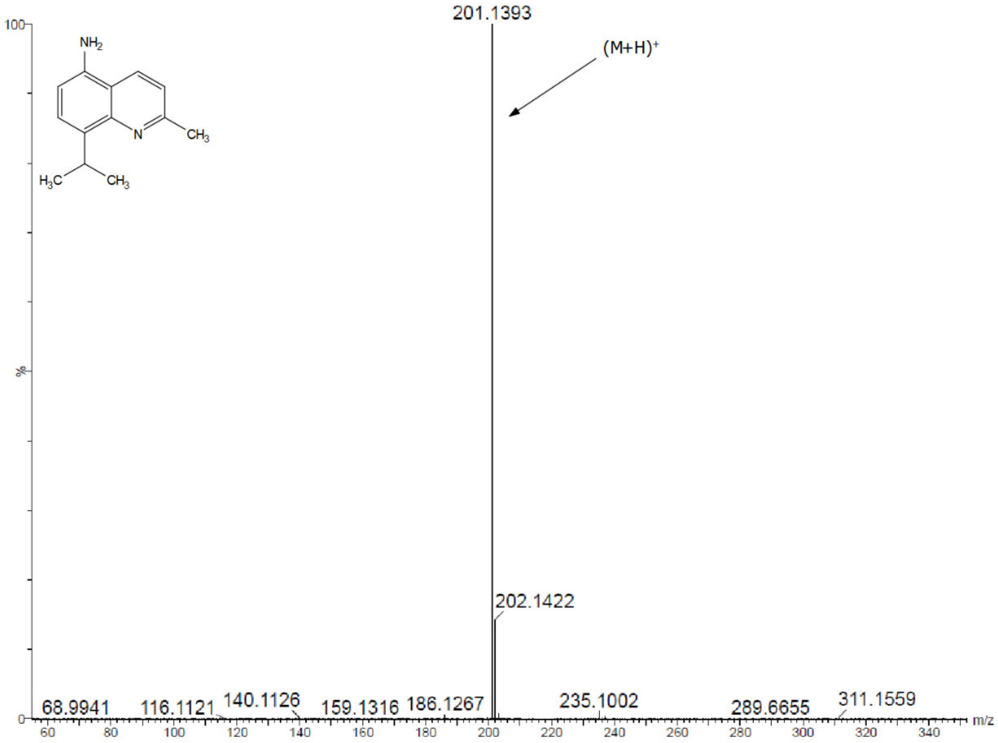


**Fig. S12e.** MS spectrum of the **6d**.
